# Time-dependent comparative efficacy of non-surgical treatments for pain relief in lateral epicondylitis: a systematic review and network meta-analysis

**DOI:** 10.3389/fphys.2026.1782562

**Published:** 2026-03-27

**Authors:** Congying Ouyang, Qiuping You, Chengbo Yang, Dawei Yu

**Affiliations:** 1 School of Sport and Training, Chengdu Sport University, Chengdu, China; 2 College of Teacher Education (Physical Education), Taizhou University, Taizhou, China

**Keywords:** lateral epicondylitis, network meta-analysis, non-surgical treatment, pain relief, time-dependent efficacy

## Abstract

**Background:**

Lateral epicondylitis (LE) is a common tendinopathy, but the relative pain-relieving efficacy of different non-surgical interventions across follow-up stages remains unclear. This study used a Bayesian network meta-analysis to compare the time-dependent effects of multiple non-surgical treatments for pain relief in LE.

**Methods:**

PubMed, Web of Science, and the Cochrane Central Register of Controlled Trials were systematically searched from database inception to March 5, 2024, with an updated search conducted to February 2, 2026. Randomized controlled trials involving adults with LE who received non-surgical interventions were included. Pain intensity measured by the visual analog scale (VAS) was the primary outcome, and all VAS scores were standardized to a 0–10 scale, with lower scores indicating less pain. Post-treatment VAS scores were synthesized in three predefined time windows: short-term (1–4 weeks; the result closest to 4 weeks), intermediate-term (4–12 weeks; the result closest to 12 weeks), and long-term (>12 weeks; the longest follow-up beyond 12 weeks). A Bayesian random-effects network meta-analysis was performed. Treatment effects were expressed as mean differences (MDs) with 95% credible intervals (CrIs), and ranking probabilities were summarized using the surface under the cumulative ranking curve (SUCRA).

**Results:**

A total of 27 randomized controlled trials were included. In the short term, kinesio taping (KT), corticosteroid injection (CSI), brace, and laser therapy (LA) showed superior pain relief compared with placebo; the MDs (95%CrIs) for KT and CSI were -4.10 (-6.14 to -2.11) and -3.57 (-5.71 to -1.47), respectively. In the intermediate term, CSI, extracorporeal shock wave therapy (ESWT), glycosaminoglycan polysulfate (GAGPS), KT, physical therapy (PT), pulsed ultrasound (PU), and ultrasound (US) were superior to placebo; the MDs (95%CrIs) for KT and CSI were -2.58 (-3.92 to -1.32) and -1.60 (-2.77 to -0.44), respectively. In the long term, no intervention showed a statistically significant advantage over placebo. Although KT and CSI ranked relatively high in the short term, and GAGPS and KT ranked relatively high in the intermediate term, ranking results should be interpreted cautiously in light of interval width, direct evidence, and network consistency.

**Conclusion:**

The pain-relieving effects of non-surgical interventions for LE appear to be time-dependent. Some treatments may be more favorable for short- or intermediate-term pain relief, but evidence for long-term superiority remains insufficient. Clinical interpretation should not rely on treatment ranking alone, but should instead integrate the follow-up stage, effect estimates, and evidence certainty. More high-quality randomized controlled trials with long-term follow-up are needed to clarify the long-term value of different non-surgical treatment strategies.

## Introduction

Lateral epicondylitis (LE), commonly known as tennis elbow, is a common elbow tendinopathy that primarily affects the common extensor tendon origin of the forearm. It typically presents as gradually worsening pain on the lateral side of the elbow, usually without a clear history of trauma (Tosti et al., 2013). LE is now widely considered to be associated with repetitive overuse and mechanical loading, leading to microinjury, collagen disorganization, and degenerative changes in tendon tissue rather than being a purely inflammatory disorder (Ahmad et al., 2013; Karbowiak et al., 2023; Wolf, 2023). Epidemiological studies have shown that LE affects approximately 1%-2% of the general population, with incidence varying across occupational and athletic populations (Shiri et al., 2006; Abrams et al., 2012; Descatha et al., 2013; Herquelot et al., 2013). Among tennis players, the prevalence may be as high as approximately 40% (Abrams et al., 2012), whereas in the general working population the annual incidence is about 1.0% in men and 0.9% in women (Herquelot et al., 2013). LE is particularly common in occupations involving repetitive forearm rotation and wrist extension, such as carpentry, construction work, floor installation, and cleaning (Descatha et al., 2013). Clinically, it is characterized by tenderness over the lateral elbow, pain radiating along the forearm, and exacerbation during gripping or wrist extension; in severe cases, it may affect daily activities and sleep (Ahmad et al., 2013).

The natural course of LE is usually self-limiting, and symptoms improve within 12–18 months in most patients (Dean et al., 2014; Wolf, 2023). However, in some cases the condition may persist for a prolonged period or recur intermittently (Vuvan et al., 2020). Therefore, how to intervene effectively at different stages to reduce pain and shorten symptom duration remains an important issue in both clinical practice and research.

At present, treatment for LE is still mainly non-surgical and includes exercise therapy, physical therapy, bracing or banding, injection therapy, and a wait-and-see approach (Bisset et al., 2006; Vuvan et al., 2020). Corticosteroid injection can provide rapid pain relief, but its long-term efficacy remains controversial, and local glucocorticoid use may have unfavorable biological effects on tendon tissue (Beyzadeoglu et al., 2011; Dean et al., 2014). In contrast, some systematic reviews suggest that platelet-rich plasma (PRP) may be superior to corticosteroids for medium- and long-term pain relief and functional improvement, whereas corticosteroids appear to have greater benefit in the short term (Beyzadeoglu et al., 2011; Xu et al., 2024). In addition, non-injection treatments such as shock wave therapy, ultrasound, manual therapy, exercise training, and bracing are widely used in LE management. Their potential effects may be related to load modulation, pain relief, and local tissue adaptation; however, the relative efficacy of these treatments at different time stages remains unclear (Vuvan et al., 2020; Liu et al., 2022; Xu et al., 2024).

However, substantial heterogeneity exists in the current literature with respect to intervention types, follow-up time points, and outcome measures (such as pain, function, and grip strength), making it difficult to directly compare the true pain-relieving effects of different treatments across stages (Kim et al., 2021; Perveen et al., 2024). Network meta-analysis can integrate direct and indirect evidence within a unified framework and thus allows a more comprehensive comparison of the relative effects of multiple treatment strategies (Hutton et al., 2015). Therefore, the present study used the visual analog scale (VAS) as the primary outcome to systematically evaluate the relative efficacy of multiple non-surgical interventions for LE pain relief across different follow-up stages (1–4 weeks, 4–12 weeks, and >12 weeks). Our aim was to clarify the time-dependent effects of different treatment strategies and to provide more reliable evidence to support stage-specific pain management and rehabilitation decision-making.

## Materials and methods

This study was a systematic review and network meta-analysis (NMA), designed and reported in accordance with the Preferred Reporting Items for Systematic Reviews and Meta-Analyses (PRISMA) statement and its extension for network meta-analyses (Hutton et al., 2015). The protocol was prospectively registered in the PROSPERO database (registration number: CRD42024532881).

### Eligibility criteria

Randomized controlled trials (RCTs) evaluating non-surgical interventions for adult patients with lateral epicondylitis (LE) were included. Eligible participants were aged 18–70 years. LE was defined as local pain over the lateral epicondyle that worsened with resisted wrist extension, gripping, or palpation of the common extensor tendon. Studies were included if they met the following criteria: (1) RCT design; (2) evaluation of at least one non-surgical intervention; (3) inclusion of a comparator group suitable for analysis; (4) pain intensity measured by the visual analog scale (VAS) as the primary outcome; and (5) extractable outcome data, including means, standard deviations, and sample sizes, or sufficient information for derivation. In this study, control groups were defined as placebo or minimal-intervention controls, including wait-and-see, sham treatment, sham injection, sham ultrasound, placebo taping, or other minimal interventions without clear therapeutic activity. Studies in which the definition of minimal intervention was unclear or which actually contained active therapeutic components were excluded. Exclusion criteria were as follows: (1) studies including patients with severe elbow trauma, prior elbow surgery, systemic inflammatory disease, or other major comorbidities likely to substantially affect pain outcomes; (2) studies including participants younger than 18 years; (3) non-randomized studies, reviews, conference abstracts, protocols, or studies without complete outcome data; (4) duplicate publications, overlapping samples, or repeated datasets; (5) studies with very high attrition (>80%) and unreliable outcome assessment; (6) combined or multimodal interventions for which the effect of a single treatment could not be separated; (7) studies comparing the same intervention only on the basis of different intensities, doses, or technical details, which were excluded to reduce clinical heterogeneity; (8) studies involving bloodletting therapy or other interventions inconsistent with the predefined treatment framework; and (9) studies with unclear outcome definitions, obviously abnormal data, or data that could not be verified.

### Information sources and search strategy

PubMed, Web of Science, and the Cochrane Central Register of Controlled Trials (CENTRAL) were systematically searched to identify randomized controlled trials evaluating non-surgical interventions for LE. The initial search covered the period from database inception to March 5, 2024. In response to reviewers’ comments, an updated search of the same three databases was conducted, with a final search date of February 2, 2026. The search strategy combined subject headings and free-text terms and focused on the following concepts: (1) disease-related terms, such as “lateral epicondylitis,” “tennis elbow,” and “lateral elbow pain”; (2) intervention-related terms, such as “physical therapy,” “exercise,” “injection,” “platelet-rich plasma,” “corticosteroid,” “bracing,” “shockwave,” and “nonoperative treatment”; and (3) study-design terms, such as “randomized controlled trial,” “randomized,” and “trial.” In addition, the reference lists of included studies and relevant reviews were manually screened to minimize the risk of missing eligible studies. After the updated search, 342 additional records were identified; 16 duplicates were removed; 297 records were excluded after title and abstract screening; 29 full texts were assessed; and 3 newly eligible studies were finally included. The full search strategies are provided in **Supplementary Material S5**.

### Study selection and data extraction

Study selection and data extraction were performed independently by two reviewers. Titles and abstracts were screened first, followed by full-text assessment of potentially eligible studies. Disagreements were resolved by discussion; if consensus could not be reached, a third reviewer made the final decision. The following information was extracted from each included study: first author, publication year, country or region, diagnostic criteria for LE, sample size, age, sex distribution, symptom duration, intervention and detailed treatment protocol, control type, follow-up time points, pain VAS outcome data, and information required for risk-of-bias assessment. All extracted data were cross-checked by the two reviewers to ensure accuracy. Post-treatment values rather than change-from-baseline values were used to calculate effect sizes. If multiple follow-up assessments were reported within the same time window, only one representative time point was extracted according to prespecified rules: for the short term, the result closest to 1–4 weeks; for the intermediate term, the result closest to 4–12 weeks; and for the long term, the longest follow-up beyond 12 weeks. If standard deviations were not directly reported, they were derived from the text, tables, or supplementary materials whenever possible. Data were independently extracted by both reviewers and then reconciled.

### Outcome measures and definition of time windows

The primary outcome of this study was pain intensity measured by the VAS. Because the included studies reported VAS on both 0–10 and 0–100 scales, all 0–100 scores were linearly converted to a 0–10 scale to ensure comparability. After conversion, lower scores indicated less pain and better treatment effect. Based on the follow-up characteristics of the included studies and clinical relevance, pain outcomes were grouped into three predefined time windows: (1) short-term: 1–4 weeks; (2) intermediate-term: >4 weeks to 12 weeks; and (3) long-term: >12 weeks. This categorization was intended to maximize comparability across studies and to reflect stage-specific changes in treatment effect.

### Risk of bias assessment

Two reviewers assessed the methodological quality of the included RCTs using the Cochrane risk-of-bias tool implemented in Review Manager. The domains evaluated included random sequence generation, allocation concealment, blinding of participants and personnel, blinding of outcome assessment, incomplete outcome data, selective reporting, and other potential sources of bias. Each domain was judged as low risk, high risk, or unclear risk according to Cochrane Handbook criteria. Disagreements were resolved by discussion, with arbitration by a third reviewer when necessary. To evaluate robustness, sensitivity analyses were later performed by excluding studies judged to have a high overall risk of bias.

### Network geometry and transitivity assessment

Network plots were first generated to illustrate the direct comparison structure between interventions. In the network plots, nodes represented interventions, node size was proportional to the total sample size receiving each intervention, and edges represented direct head-to-head comparisons, with edge thickness proportional to the number of studies contributing to that comparison. A contribution matrix was also constructed to evaluate the relative contribution of each direct comparison to the overall network estimates. Before conducting the network meta-analysis, we assessed the plausibility of the transitivity assumption, that is, whether the distribution of potential effect modifiers was broadly similar across treatment comparisons. Prespecified potential effect modifiers included mean age, sex proportion, baseline VAS score, symptom duration, and major intervention characteristics. These baseline features were summarized and compared to judge whether there were obvious clinical or methodological differences that might threaten the validity of indirect comparisons.

### Statistical analysis

All statistical analyses were conducted using the MetaInsight platform. Because all pain outcomes were standardized to a common 0–10 VAS scale, mean difference (MD) was used as the effect measure, with 95% credible intervals (CrIs) reported. The direction of effect was defined such that MD < 0 indicated that the former intervention was more favorable than the latter for pain reduction. A Bayesian random-effects network meta-analysis model was used to synthesize direct and indirect evidence under the assumption of transitivity. Separate treatment networks were constructed for each predefined time window: short-term (1–4 weeks), intermediate-term (4-12weeks to 12 weeks), and long-term (>12 weeks). For each time window, relative treatment effects and corresponding 95%CrIs were calculated. To evaluate the relative ranking of interventions, ranking probabilities were estimated and summarized using the surface under the cumulative ranking curve (SUCRA). SUCRA ranges from 0% to 100%, with higher values indicating a greater probability of being among the more favorable treatments for pain relief. However, ranking results were interpreted only as relative probabilities and were considered in conjunction with effect estimates, the width of the 95%CrIs, and the adequacy of direct evidence.

### Consistency assessment, heterogeneity, and sensitivity analyses

Internal consistency of the network was assessed using node-splitting and inconsistency factors (IFs) within closed loops. A node-splitting *p* value > 0.05 or an IF 95%CrI including 0 was considered evidence of no statistically significant inconsistency between direct and indirect evidence. In addition to overall heterogeneity assessment, the uncertainty of ranking results was interpreted cautiously with reference to network structure, the number of direct comparisons, and the width of credible intervals. Sensitivity analyses were performed to test the robustness of the findings, mainly by excluding studies with a high overall risk of bias or with clear clinical or methodological heterogeneity and then comparing changes in effect estimates and treatment ranking across time windows. These analyses were used to further evaluate the stability of intervention ranking over time and to reduce potential interpretive bias arising from sparse evidence, excessive reliance on indirect evidence, or network imbalance.

## Results

### Characteristics of included studies

**Figure 1:**
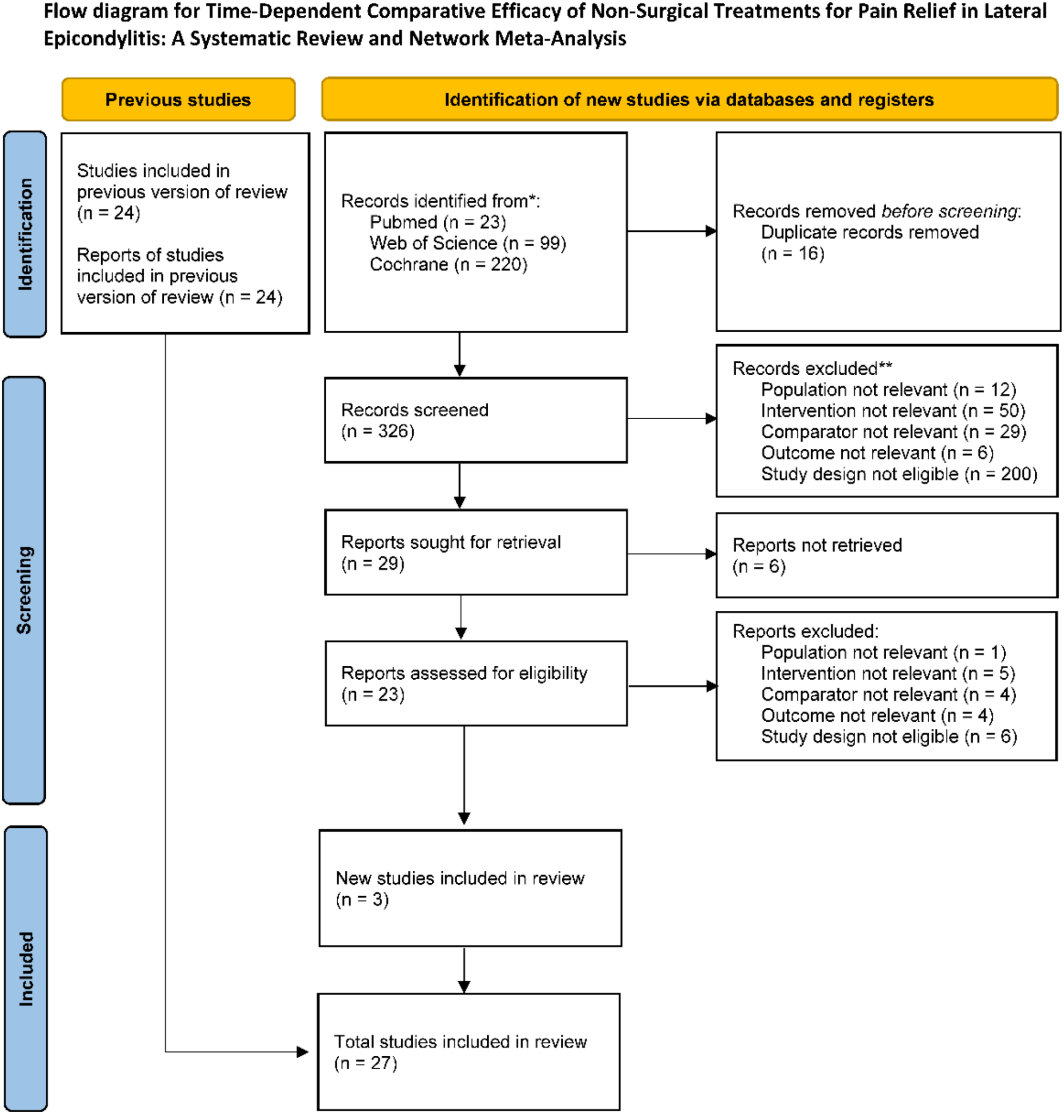
Flow diagram of study selection. The figure illustrates the identification, screening, full-text assessment, and final inclusion of studies in the updated review. Based on the 24 studies included in the previous version of the systematic review, 3 additional studies were identified through the updated search, resulting in a total of 27 included studies.

The basic characteristics of the included studies are summarized in **Table 1**. A total of 27 randomized controlled trials were ultimately included (Akermark et al., 1995; Rompe et al., 2002; Wong et al., 2005; Bisset et al., 2006; Lam and Cheing, 2007; Ceyda et al., 2010; Blanchette and Normand, 2011; Cherry et al., 2012; Gündüz et al., 2012; Küçükşen et al., 2013; Devrimsel et al., 2014; Dundar et al., 2015; Montalvan et al., 2016; Aydin and Atiç, 2018; Creuzé et al., 2018; Yalvaç et al., 2018; Yerlikaya et al., 2018; Baktir et al., 2019; Koçak et al., 2019; Akcay et al., 2020; Çorum et al., 2021; Özmen et al., 2021; Hüseyin Ünver et al., 2021; Agarwal et al., 2023; Akkurt et al., 2025; Hoseini et al., 2025; Kizilkurt et al., 2025), published between 1995 and 2025, of which 24 were from the previous version of the systematic review and 3 were newly included after the updated search. Most studies reported explicit diagnostic criteria for lateral epicondylitis, and all participants were adults. Pain intensity assessed by the visual analog scale (VAS) was used as the primary outcome in all studies. Sample sizes varied substantially across studies, although most were small to moderate in size. The mean age of participants was generally between 30 and 50 years, and the proportion of female participants was overall higher than that of males. Most studies enrolled patients with a symptom duration longer than 3 months, although some studies did not report this information. The interventions covered a wide range of non-surgical approaches, including placebo or wait-and-see controls, physical therapy, exercise therapy, extracorporeal shock wave therapy, ultrasound therapy, laser therapy, manual therapy, supportive treatments such as taping or bracing, and injection therapies including corticosteroids, dextrose prolotherapy, platelet-rich plasma, and botulinum toxin type A. Most studies used a two-arm parallel-group design, while some used three-arm comparisons. Follow-up duration ranged from 2 to 52 weeks and was stratified into short-term (1–4 weeks), intermediate-term (4 weeks to 12 weeks), and long-term (>12 weeks). The study selection process is shown in **Figure 1**.

**Table 1:** Intervention-related characteristics of the included randomized controlled trials.

Study	Diagnosis reported	Duration (month)	Sample size (F/M)	Mean age (years)	Intervention details	Outcome measure	Follow-up (Weeks)	Main result
Agarwal et al., 2023	Yes	NR	10 10	30-45	HVLAT: high velocity low amplitude thrust Physiotherapy: 3 sessions/week on alternative days for 1 week	VAS	24	HVLAT^+^
Agostinucci et al., 2012	Yes	>3 >3	74	NR	EX: traditional exercise Cryo-Max®: three cycles of 20 minutes cold pack	VAS	6	Cryo-Max®+
Akcay et al., 2020	Yes	9 18	23 (18/5) 27 (19/8)	48.1 46.7	DPT: 15% dextrose, 1.5 mL Placebo: 1.5 mL, no peppering	VAS	0, 4, 8, 12	Saline+
Akermark et al.,1995	Yes	3-36 3-30	30 (15/15) 30 (14/16)	46 42	GAGPS: 50 rng, one/week, five weeks Placebo: 50 rng, one/week, five weeks	VAS	3, 2, 12, 26	GAGPS+
Akin et al., 2010	Yes	8.7 6.8	30 (18/12) 30 (20/10)	46.7 45.5	Ultrasound: 15 US, Petas Petson 250 US 2200 for 1 MHz and 1.5 W/cm2 Placebo: nonworking head for 5 minutes	VAS	3, 5	US+
Aydin et al., 2018	Yes	1 1	32 (17/15) 35 (16/19)	38.84 37.94	ESWT: 4 sessions per week, 10–12 Hz, 2,000 pulses, and 1.6-1.8 bar pressure WES: a wrist splint at 30°–45° extension for 4 weeks	VAS	4, 12, 24	WES+
Baktir et al., 2019	Yes	11 12.25 12	12 (10/2) 12 (9/3) 13 (10/3)	45.33 43.75 49.31	Laser: 904 nm wavelength of, 57000 Hz, peak power of 27 W, 50 W, or 27×4W Phonophoresis: 2 mg/d prednisolone, 5 cm2 US head, 1 W/cm2 dosage, 1 MHz, 7 minutes Iontophoresis: 5 mL of 0.4% prednisolone	VAS	3	Iontophoresis+
Bisset et al., 2006	Yes	6.06 6.06 3.7	67 (24/43) 65 (25/40) 66 (21/45)	47.3 47.8 47.9	WAS: wait and see CI: 1 ml of 1% lidocaine with 10 mg of triaminolone acetonide in 1 ml PT: eight treatments of 30 minutes, 6 weeks	VAS	3, 6, 12, 26, 52	CI+
Blanchette et al., 2011	Yes	22 43	15 (9/6) 12 (6/6)	47 46	ASTM: 2 times/week/5 weeks Control: extensors muscles of the wrist (hold 30 seconds, 6 times a day)	VAS	6, 12	No Difference
Çorum et al., 2021	Yes	>3 >3	22 (16/6) 19 (14/5)	49 45	ESWT: once per week with 1.8 bar pressure, 10 Hz, 2,000 pulses. Exercise: 3 times/week for three weeks	VAS	4, 12	No Difference
Creuze et al., 2018	Yes	17.2 20.2	30 (13/17) 30 (14/16)	47.3 46.7	BoNT-A: 40 IU of BoNT-A from a 500-IU flask, diluted in 5 mL of saline solution Placebo: 0.4 mL of saline solution	VAS	12	BoNT-A+
Devrimsel et al., 2014	Yes	NR	30 (22/8) 30 (20/10)	37.76 40.3	ESWT: 2000 shock waves, 1.6 bar intensity and 16 Hz, 3 times/3 weeks, a 1-week interval Laser: 10 sessions with 3.6 joule intensity, 500 Hz, and 850 nm wavelength,	VAS	12	ESWT+
Dundar et al., 2015	Yes	28.7 29.5 27.9	30 (17/13) 31 (17/14) 30 (15/15)	32.6 33.4 33.6	HILT: 360–1780 mJ/cm2, 120-150 μs, 10.5 W, 10–40 Hz Placebo: shame placebo Brace: lateral counterforce brace	VAS	4, 12	HILT+
Gündüz et al., 2012	Yes	3 3 3	19 (14/5) 20 (12/8) 20 (12/8)	43.6 45.7 44.9	PT: US 1 W/cm2, 5 min, friction massage 5 min for ten sessions CI: 20 mg methylprednisolone acetate and 1 ml prilocaine ESWT: pressure 1.4 bar, frequency 4.0 Hz, number 500 for ten sessions	VAS	4. 12. 24	ESWT+
Huseyin et al., 2021	Yes	3.5 3.7 3.7	17 (10/7) 17 (12/5) 17 (11/6)	45.3 47.2 47.1	CUS: 1.5 MHz and 1 W/cm2 for 5 min per session PUS: 1.5 MHz and 1 W/cm2 a pulsed mode duty cycle of 1:4 Placebo US: received a sham US	VAS	6	CUS+ PUS+
Koçak et al., 2019	Yes	4.50 5.07	28 (14/14) 28 (18/10)	43.54 40.96	SI: 20 mg of methylprednisolone acetate (0.5 mL) and 0.5 mL of prilocaine at 2% KT: tape treatment with a 15%-25% stretch, no stretching	VAS	3, 12	KT+
Küçüksen et al., 2013	Yes	5.3 6.1	41 (23/18) 41 (22/19)	46.17 43.78	MET: forearm pronation and supination in 8 sessions, each with 5 repetitions of 5-second contractions CSI: 1mL of triamcinolone (40mg/mL) and 1mL of lidocaine (10mg/mL)	VAS	6, 26, 52	MET+
Lam et al., 2007	Yes	3.2 3.3	21 (12/9) 18 (11/7)	46.1 48.9	LA: 5000 Hz, 2.4 J/cm², 11 sec per point; 0.66 J for 2.4 tender points Placebo: sham irradiation	VAS	3	LA+
Montalvan et al., 2016	Yes	≤3	50	35-65	PRP: 12 ml blood; PRP showed 1.6x platelet enrichment, with 2 ml syringes for PRP and saline. Placebo: 2 ml of 1% lidocaine s.c.	VAS	12	No Difference
Özmen et al., 2021	Yes	2.92 7 8.07	13 (6/7) 13 (8/5) 14 (10/4)	49.62 47.15 48.36	US: hot pack, transcutaneous electrical nerve stimulation, and US therapy KT: hot pack, TENS ESWT: hot pack, TENS	VAS	2, 8	KT+
Rompe et al., 2003	Yes	15.9 12	40 (21/19) 35 (21/14)	46.5 48.2	ESWT: 1500 pulses at 0.18 mJ/mm2 Placebo: sham treatments	VAS	12	ESWT+
Wong et al., 2005	Yes	11.83 19.07	30 (25/5) 30 (24/6)	45.60 44.18	BoNT-A: 60 units Placebo: an equivalent volume of normal saline	VAS	4, 12	BoNT-A+
Yalvaç et al., 2018	Yes	7.9 8.2	24 (16/8) 20 (15/5)	43.75 46.04	US: 1 cm2 application area, at 1.5 W/cm2, 1 MHz frequency, ESWT: 10–15 Hz, 1.5-2.5 bar energy, 2000 pulses	VAS	4	ESWT+
Yerlikaya et al., 2018	Yes	>3	30 (19/11) 30 (26/4) 30 (19/11)	47.6 45.0 46.5	Control: 1.5 ml saline LP-PRP1: 1.5 ml saline LR-PRP2: 1.5 ml saline	VAS	4, 8	No Difference
Hoseini et al., 2025	Yes	5.2 5.5 5.5	17 (12/5) 17 (11/6) 17 (9/8)	43.58 45 42	Brace: Y-shaped Kinesio strips Kinesio Taping: 1 ml of triamcinolone (20 mg/ml), 1 ml of 1% lidocaine Cortico steroid: Counterforce brace worn continuously for two weeks	VAS	4	All+
Akkurt et al., 2025	Yes	7 12	21 (9/12) 21 (8/13)	43.0 46.14	KT Group: A standard 5-cm wide Kinesio® Tex Gold Sham Group: non-allergic, non-elastic medical cloth tape	VAS	3, 7	KT+
Kizilkurt et al., 2025	Yes	> 3	12 (7/5) 12 (6/6) 12 (5/7)	42.2 43.4 40.7	Corticosteroid: 2 mL total (1 mL betamethasone + 1 mL bupivacaine HCl) PRP: 2 mL PRP (from 16.2 mL blood + 1.8 mL sodium citrate)	VAS	12, 24	PRP+

“Diagnosis reported” indicates whether explicit diagnostic criteria for lateral epicondylitis were provided in the original study. Yes = reported; NR = not reported. F/M = female/male. Symptom duration is presented in months and follow-up in weeks. In the “Main result” column, “+” indicates that the intervention listed showed a more favorable pain-relieving effect than its comparator at the reported follow-up time point; “No difference” indicates that no statistically significant between-group difference was reported; “All” indicates improvement in all study arms without a clearly superior intervention. VAS, visual analog scale; NR, not reported; HVLAT, high-velocity low-amplitude thrust; EX, exercise; DPT, dextrose prolotherapy; GAGPS, glycosaminoglycan polysulfate; US, ultrasound; CUS, continuous ultrasound; PUS, pulsed ultrasound; ESWT, extracorporeal shock wave therapy; WES, wrist extension splint; WAS, wait-and-see; PT, physical therapy; CI, corticosteroid injection; SI, steroid injection; CSI, corticosteroid injection; ASTM, augmented soft tissue mobilization; KT, kinesio taping; MET, muscle energy technique; LLLT, low-level laser therapy; HILT, high-intensity laser therapy; BoNT-A, botulinum toxin A; PRP, platelet-rich plasma; LP-PRP, leukocyte-poor platelet-rich plasma; LR-PRP, leukocyte-rich platelet-rich plasma.

### Risk of bias assessment

**Figure 2:**
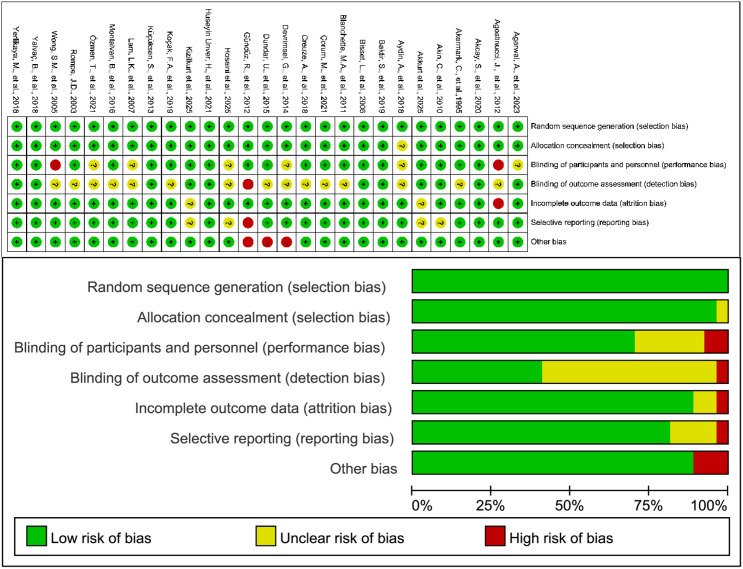
Risk of bias assessment of the included studies. The upper panel presents the risk of bias judgments for each included study across the seven domains of the Cochrane risk of bias tool, while the lower panel summarizes the overall proportion of studies rated as low, unclear, or high risk of bias in each domain.

The results of the risk-of-bias assessment are shown in **Figure 2**. Overall, most included studies were judged to have a low risk of bias across most domains. Random sequence generation was rated as low risk in all studies, and allocation concealment was also predominantly judged as low risk, with only a few studies rated as unclear because of insufficient reporting. By contrast, blinding-related domains showed more unclear or high-risk judgments, particularly for blinding of outcome assessment, where the proportion of unclear risk was relatively high. Incomplete outcome data and selective reporting were mostly assessed as low risk, although a few studies were judged as unclear or high risk. Other sources of bias were uncommon overall, with only isolated studies rated as high risk. In general, the methodological quality of the included studies was acceptable, and the main potential sources of bias were insufficient reporting of blinding procedures and outcome assessment methods.

### Short-term outcomes

**Figure 3:**
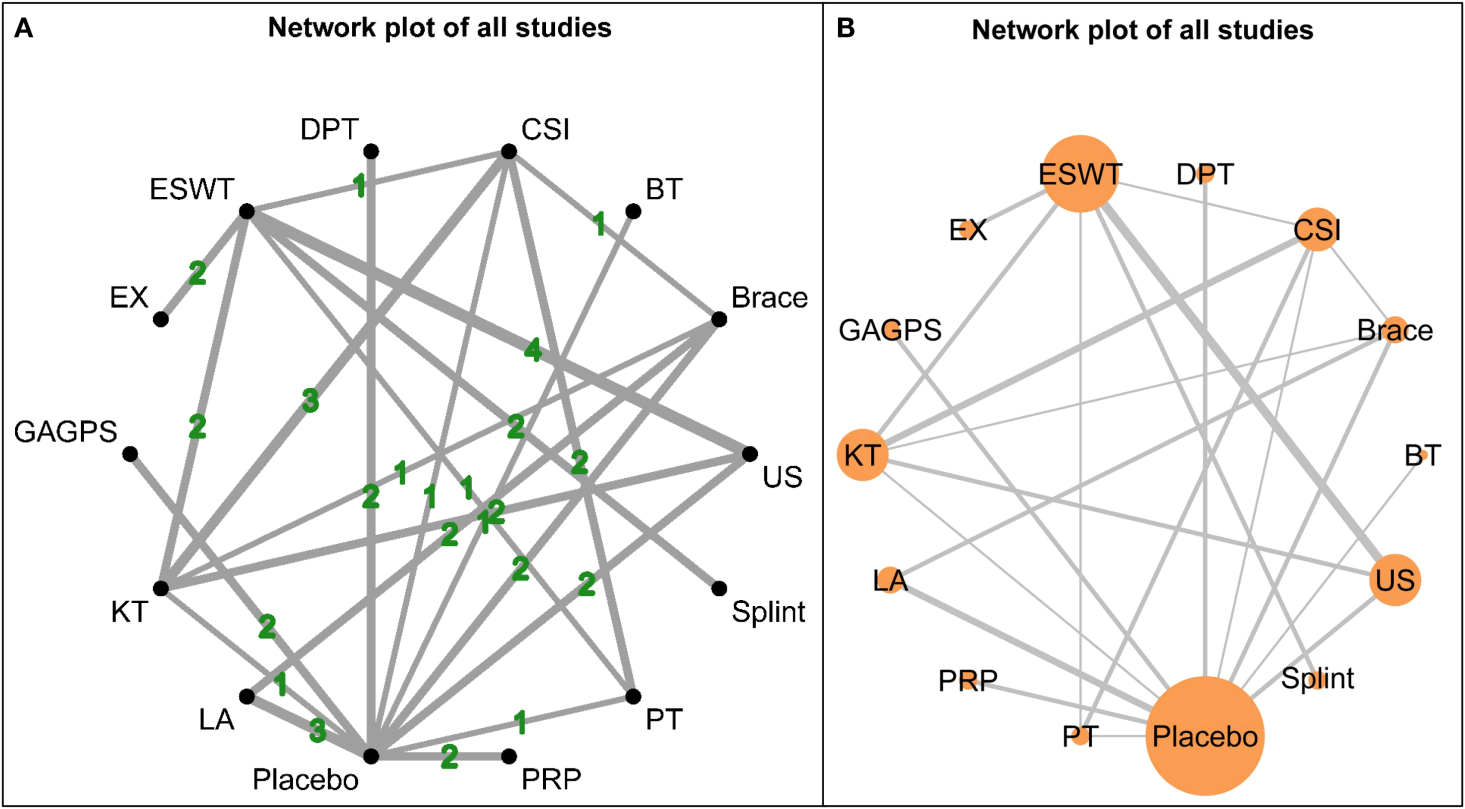
Network plots for short-term outcomes (1-4 weeks). **(A)** shows the network structure weighted by the number of participants assigned to each intervention, with node size proportional to sample size. **(B)** shows the network structure weighted by the number of direct comparisons, with edge thickness proportional to the number of studies contributing to each comparison.

The network structure for short-term outcomes is shown in **Figures 3A, B**. Fourteen interventions were included in this time window, and the overall network was well connected, with placebo serving as the main common comparator. Baseline characteristics relevant to the assessment of transitivity are presented in **Supplementary Table 1**.

**Figure 4:**
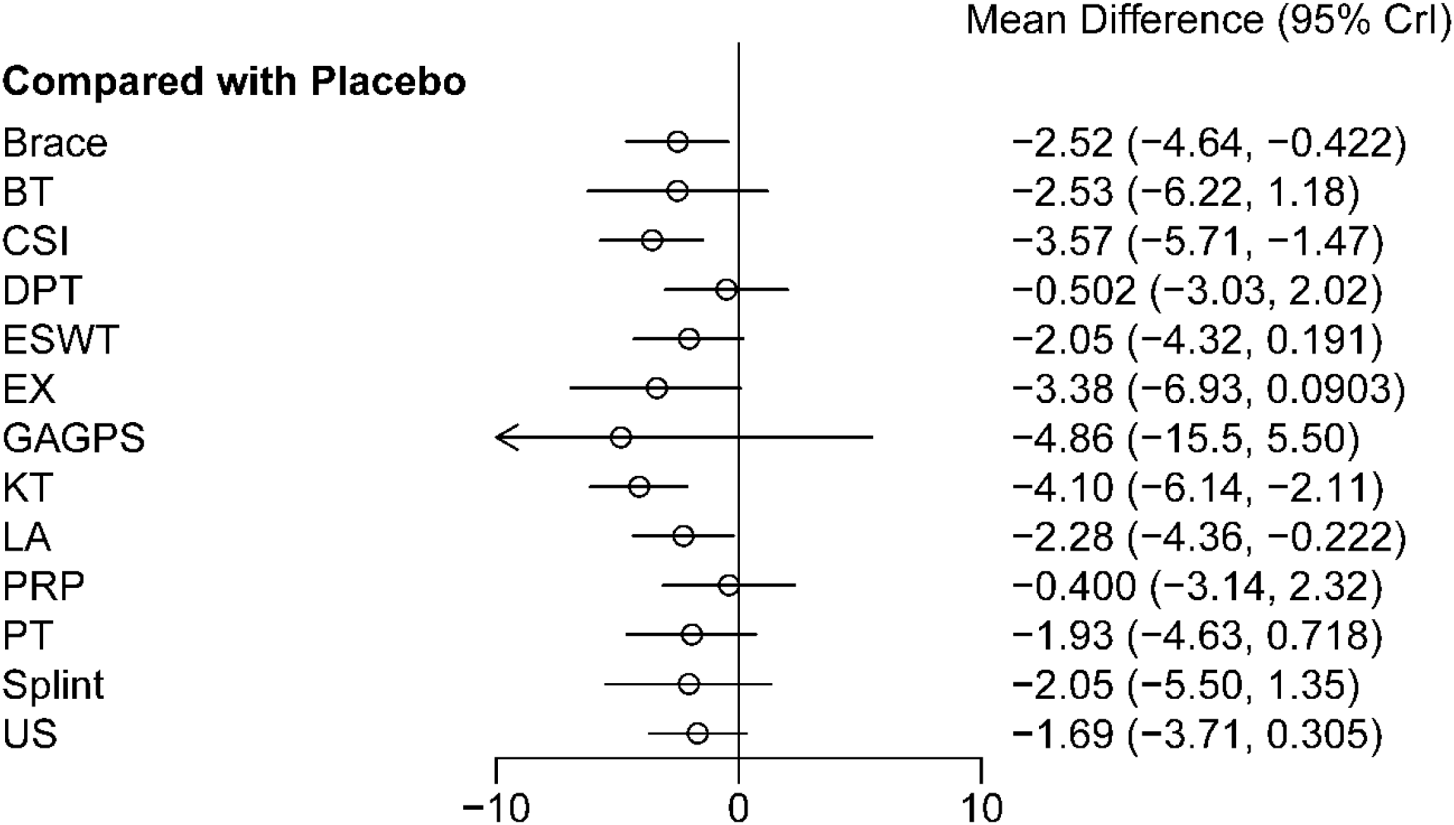
Forest plot of short-term treatment effects on pain relief (1-4 weeks). The figure presents the relative effects of each intervention compared with placebo for short-term pain relief, expressed as mean differences (MDs) with 95% credible intervals (CrIs). Negative MD values indicate greater pain reduction and therefore a more favorable treatment effect.

**Figure 5:**
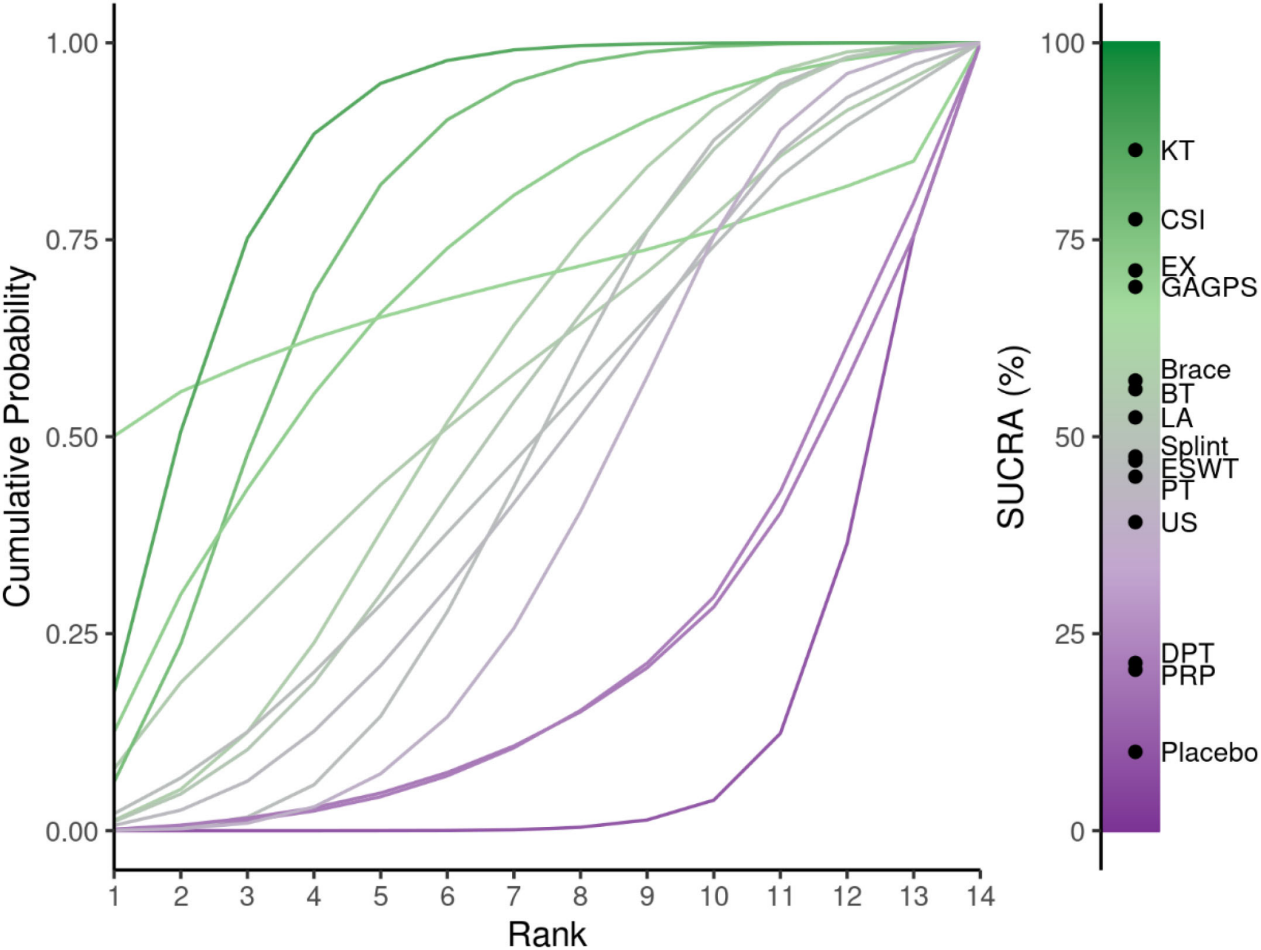
Cumulative ranking probability curves and SUCRA values for short-term pain relief (1-4 weeks). The cumulative ranking curves illustrate the probability of each intervention occupying each possible rank for short-term pain relief. SUCRA values summarize the overall ranking performance of each treatment, with higher values indicating a greater likelihood of better pain-relieving efficacy.

Forest plot results (**Figure 4**) showed that, compared with placebo, CSI, KT, brace, and LA were associated with statistically significant short-term pain relief, with MDs (95%CrIs) of -3.57 (-5.71 to -1.47), -4.10 (-6.14 to -2.11), -2.52 (-4.64 to -0.42), and -2.28 (-4.36 to -0.22), respectively. The remaining interventions did not differ significantly from placebo. Pairwise comparisons between interventions are shown in **Table 2**. The cumulative ranking probability curves are shown in **Figure 5**, and ranking probabilities and SUCRA values are reported in **Supplementary Table 2**. KT and CSI ranked relatively high for short-term pain relief, with SUCRA values of 86.39% and 77.61%, respectively. Although EX and GAGPS also had relatively high SUCRA values, their 95%CrIs versus placebo crossed the line of no effect. Placebo ranked lowest (SUCRA = 10.01%).

**Table 2:** League table of network meta-analysis results for short-term pain relief (1–4 weeks).

Intervention	Brace	BT	CSI	DPT	ESWT	EX	GAGPS	KT	LA	Placebo	PRP	PT	Splint	US
Brace	Brace	-0.01 (-4.26, 4.23)	-1.07 (-3.57, 1.45)	2.01 (-1.24, 5.31)	0.47 (-2.3, 3.22)	-0.86 (-4.74, 2.97)	-2.34 (-13.09, 8.22)	-1.59 (-4.06, 0.86)	0.24 (-2.07, 2.55)	2.52 (0.42, 4.64)	2.12 (-1.34, 5.56)	0.59 (-2.57, 3.71)	0.46 (-3.31, 4.21)	0.82 (-1.81, 3.47)
BT	0.01 (-4.23, 4.26)	BT	-1.05 (-5.33, 3.21)	2.02 (-2.47, 6.54)	0.49 (-3.88, 4.77)	-0.85 (-6.02, 4.23)	-2.33 (-13.56, 8.64)	-1.58 (-5.81, 2.63)	0.24 (-3.98, 4.49)	2.53 (-1.18, 6.22)	2.11 (-2.45, 6.71)	0.59 (-4, 5.14)	0.49 (-4.58, 5.46)	0.83 (-3.4, 5.05)
CSI	1.07 (-1.45, 3.57)	1.05 (-3.21, 5.33)	CSI	3.07 (-0.22, 6.39)	1.53 (-0.69, 3.73)	0.2 (-3.32, 3.66)	-1.3 (-12.07, 9.35)	-0.53 (-2.33, 1.25)	1.3 (-1.5, 4.1)	3.57 (1.47, 5.71)	3.18 (-0.28, 6.64)	1.64 (-0.82, 4.1)	1.52 (-1.87, 4.91)	1.89 (-0.36, 4.14)
DPT	-2.01 (-5.31, 1.24)	-2.02 (-6.54, 2.47)	-3.07 (-6.39, 0.22)	DPT	-1.54 (-4.97, 1.8)	-2.88 (-7.2, 1.41)	-4.36 (-15.26, 6.28)	-3.6 (-6.85, -0.41)	-1.78 (-5.03, 1.44)	0.5 (-2.02, 3.03)	0.1 (-3.61, 3.76)	-1.43 (-5.15, 2.2)	-1.55 (-5.84, 2.66)	-1.19 (-4.43, 2.02)
ESWT	-0.47 (-3.22, 2.3)	-0.49 (-4.77, 3.88)	-1.53 (-3.73, 0.69)	1.54 (-1.8, 4.97)	ESWT	-1.33 (-4.04, 1.36)	-2.81 (-13.67, 7.84)	-2.05 (-4.04, -0.05)	-0.23 (-3.17, 2.72)	2.05 (-0.19, 4.32)	1.66 (-1.9, 5.17)	0.12 (-2.61, 2.83)	-0.01 (-2.57, 2.57)	0.36 (-1.32, 2.06)
EX	0.86 (-2.97, 4.74)	0.85 (-4.23, 6.02)	-0.2 (-3.66, 3.32)	2.88 (-1.41, 7.2)	1.33 (-1.36, 4.04)	EX	-1.49 (-12.72, 9.45)	-0.73 (-4.07, 2.65)	1.09 (-2.85, 5.11)	3.38 (-0.09, 6.93)	2.98 (-1.44, 7.44)	1.44 (-2.35, 5.29)	1.32 (-2.37, 5.04)	1.68 (-1.48, 4.9)
GAGPS	2.34 (-8.22, 13.09)	2.33 (-8.64, 13.56)	1.3 (-9.35, 12.07)	4.36 (-6.28, 15.26)	2.81 (-7.84, 13.67)	1.49 (-9.45, 12.72)	GAGPS	0.74 (-9.81, 11.58)	2.57 (-7.96, 13.38)	4.86 (-5.5, 15.48)	4.46 (-6.22, 15.37)	2.93 (-7.76, 13.88)	2.79 (-8.12, 13.93)	3.17 (-7.4, 13.97)
KT	1.59 (-0.86, 4.06)	1.58 (-2.63, 5.81)	0.53 (-1.25, 2.33)	3.6 (0.41, 6.85)	2.05 (0.05, 4.04)	0.73 (-2.65, 4.07)	-0.74 (-11.58, 9.81)	KT	1.82 (-0.87, 4.56)	4.1 (2.11, 6.14)	3.7 (0.35, 7.1)	2.17 (-0.55, 4.88)	2.05 (-1.21, 5.32)	2.41 (0.46, 4.39)
LA	-0.24 (-2.55, 2.07)	-0.24 (-4.49, 3.98)	-1.3 (-4.1, 1.5)	1.78 (-1.44, 5.03)	0.23 (-2.72, 3.17)	-1.09 (-5.11, 2.85)	-2.57 (-13.38, 7.96)	-1.82 (-4.56, 0.87)	LA	2.28 (0.22, 4.36)	1.88 (-1.53, 5.32)	0.35 (-2.94, 3.61)	0.22 (-3.69, 4.1)	0.58 (-2.17, 3.39)
Placebo	-2.52 (-4.64, -0.42)	-2.53 (-6.22, 1.18)	-3.57 (-5.71, -1.47)	-0.5 (-3.03, 2.02)	-2.05 (-4.32, 0.19)	-3.38 (-6.93, 0.09)	-4.86 (-15.48, 5.5)	-4.1 (-6.14, -2.11)	-2.28 (-4.36, -0.22)	Placebo	-0.4 (-3.14, 2.32)	-1.93 (-4.63, 0.72)	-2.05 (-5.5, 1.35)	-1.69 (-3.71, 0.3)
PRP	-2.12 (-5.56, 1.34)	-2.11 (-6.71, 2.45)	-3.18 (-6.64, 0.28)	-0.1 (-3.76, 3.61)	-1.66 (-5.17, 1.9)	-2.98 (-7.44, 1.44)	-4.46 (-15.37, 6.22)	-3.7 (-7.1, -0.35)	-1.88 (-5.32, 1.53)	0.4 (-2.32, 3.14)	PRP	-1.54 (-5.4, 2.27)	-1.66 (-6.01, 2.68)	-1.3 (-4.66, 2.09)
PT	-0.59 (-3.71, 2.57)	-0.59 (-5.14, 4)	-1.64 (-4.1, 0.82)	1.43 (-2.2, 5.15)	-0.12 (-2.83, 2.61)	-1.44 (-5.29, 2.35)	-2.93 (-13.88, 7.76)	-2.17 (-4.88, 0.55)	-0.35 (-3.61, 2.94)	1.93 (-0.72, 4.63)	1.54 (-2.27, 5.4)	PT	-0.12 (-3.86, 3.63)	0.24 (-2.55, 3.06)
Splint	-0.46 (-4.21, 3.31)	-0.49 (-5.46, 4.58)	-1.52 (-4.91, 1.87)	1.55 (-2.66, 5.84)	0.01 (-2.57, 2.57)	-1.32 (-5.04, 2.37)	-2.79 (-13.93, 8.12)	-2.05 (-5.32, 1.21)	-0.22 (-4.1, 3.69)	2.05 (-1.35, 5.5)	1.66 (-2.68, 6.01)	0.12 (-3.63, 3.86)	Splint	0.36 (-2.68, 3.43)
US	-0.82 (-3.47, 1.81)	-0.83 (-5.05, 3.4)	-1.89 (-4.14, 0.36)	1.19 (-2.02, 4.43)	-0.36 (-2.06, 1.32)	-1.68 (-4.9, 1.48)	-3.17 (-13.97, 7.4)	-2.41 (-4.39, -0.46)	-0.58 (-3.39, 2.17)	1.69 (-0.3, 3.71)	1.3 (-2.09, 4.66)	-0.24 (-3.06, 2.55)	-0.36 (-3.43, 2.68)	US

Results are expressed as mean differences (MDs) with 95% credible intervals (CrIs) for all pairwise comparisons. All pain scores were converted to a 0–10 VAS scale. Negative MD values favor the intervention listed in the row, indicating greater pain relief compared with the intervention listed in the column. Statistically significant comparisons are those with 95%CrIs excluding 0.

**Figure 6:**
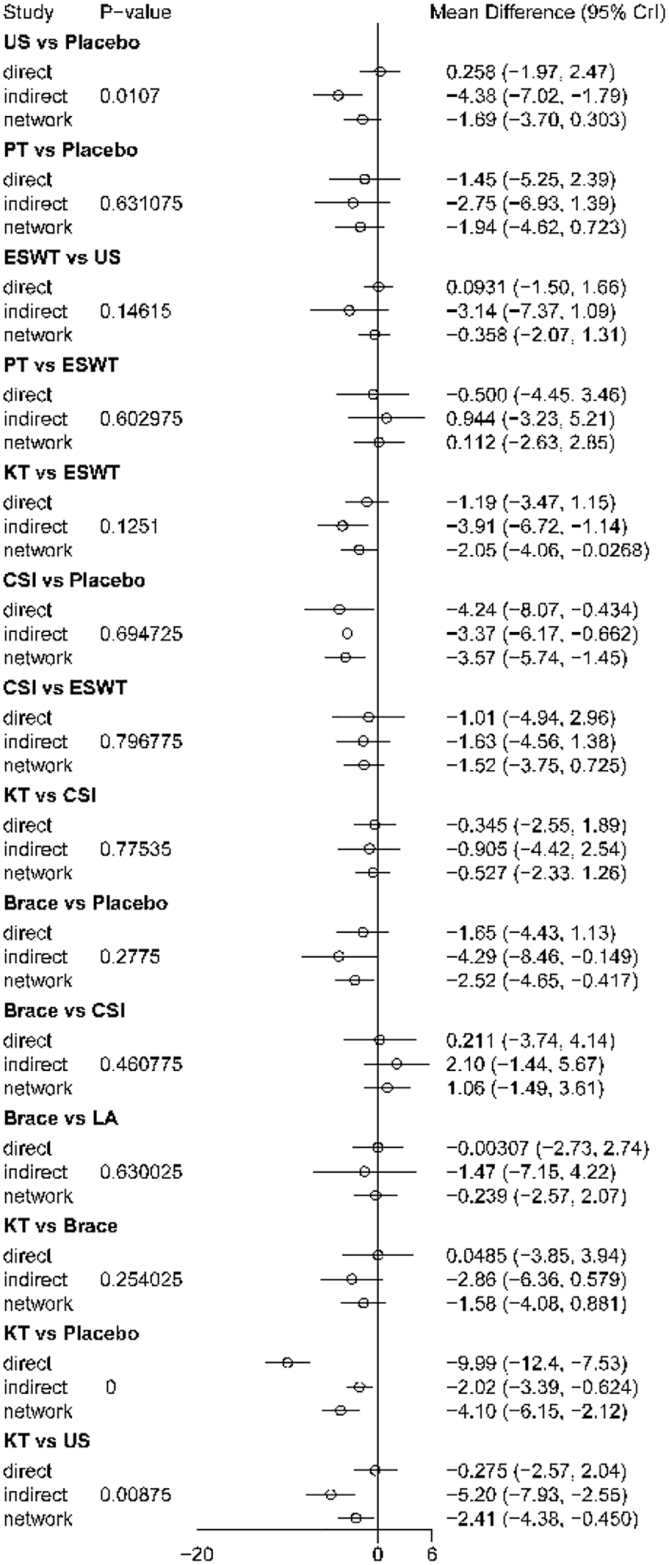
Node-splitting analysis for short-term outcomes (1-4 weeks). The figure presents the results of the node-splitting analysis used to assess local inconsistency between direct and indirect evidence in the short-term network. Comparisons with non-significant differences indicate no evidence of important inconsistency between direct and indirect estimates.

Node-splitting results are shown in **Figure 6**. Most comparisons showed no significant inconsistency; however, significant local inconsistency was detected for US versus placebo, KT versus placebo, and KT versus US, indicating discrepancies between direct and indirect evidence for some short-term comparisons. Therefore, both the short-term ranking and the corresponding effect estimates should be interpreted with caution.

### Intermediate-term outcomes

**Figure 7:**
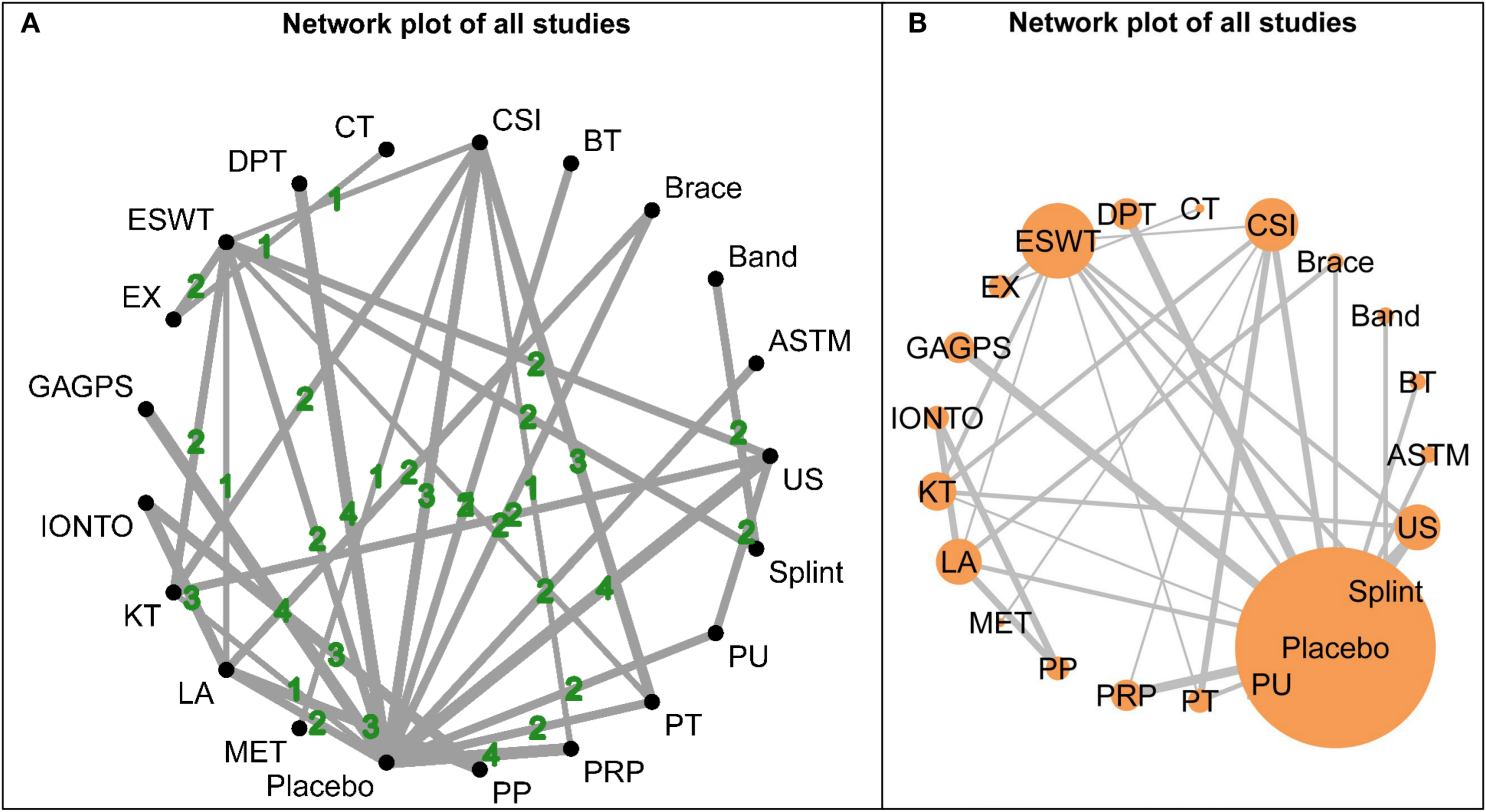
Network plots for intermediate-term outcomes (4-12 weeks). **(A)** shows the network structure weighted by the number of direct comparisons, with edge thickness proportional to the number of studies contributing to each comparison. **(B)** shows the network structure weighted by the number of participants assigned to each intervention, with node size proportional to sample size.

The network structure for intermediate-term outcomes is shown in **Figure 7A, B**. Twenty-one interventions were included in this time window, and the overall network was well connected, with placebo as the main common comparator. Baseline characteristics relevant to the assessment of transitivity are presented in **Supplementary Table 1**.

**Figure 8:**
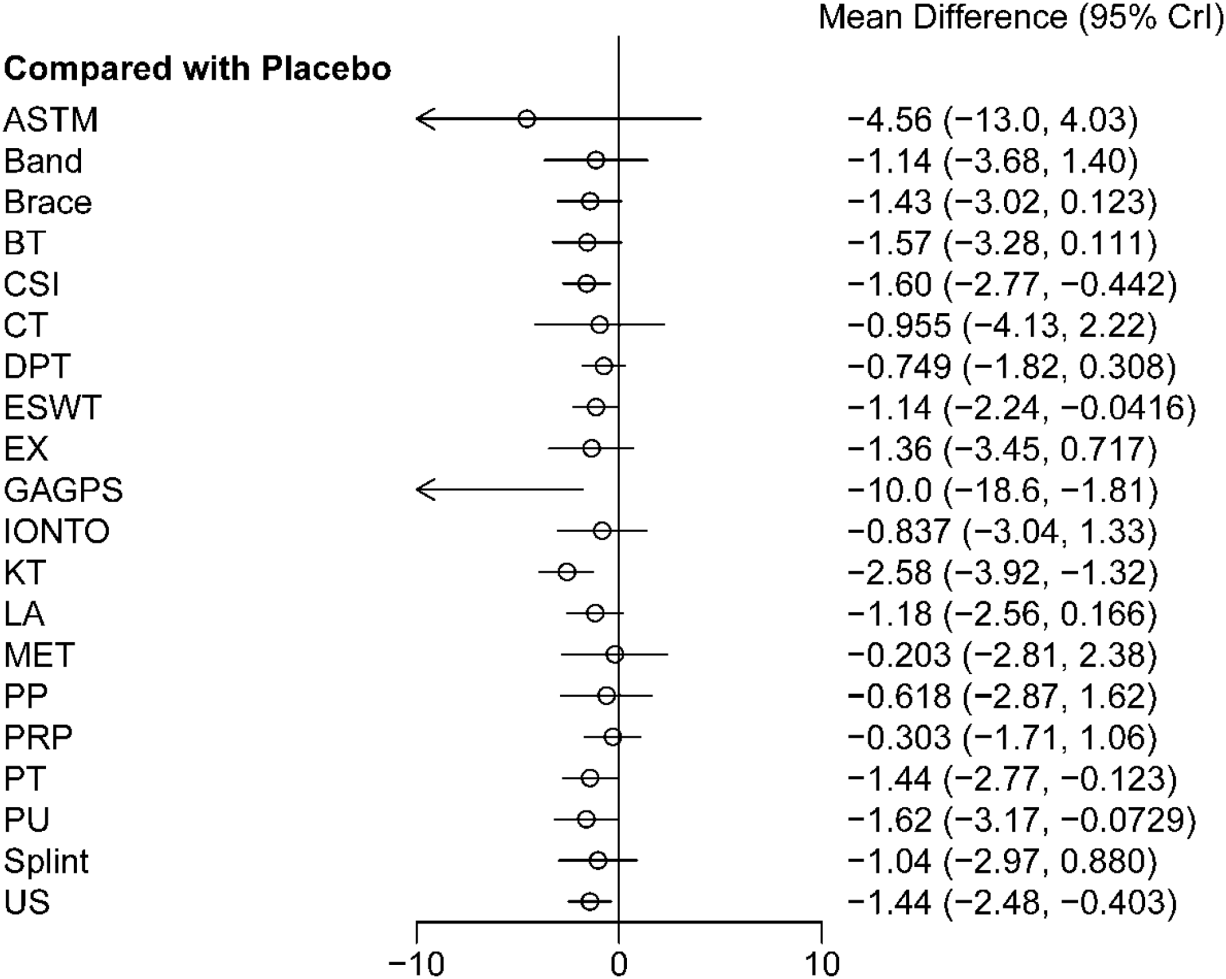
Forest plot of intermediate-term treatment effects on pain relief (4-12 weeks). The figure presents the relative effects of each intervention compared with placebo for intermediate-term pain relief, expressed as mean differences (MDs) with 95% credible intervals (CrIs). Negative MD values indicate greater pain reduction and therefore a more favorable treatment effect.

Forest plot results (**Figure 8**) showed that, compared with placebo, CSI, ESWT, GAGPS, KT, PT, PU, and US were associated with statistically significant intermediate-term pain relief, with MDs (95%CrIs) of -1.60 (-2.77 to -0.442), -1.14 (-2.24 to -0.0416), -10.0 (-18.6 to -1.81), -2.58 (-3.92 to -1.32), -1.44 (-2.77 to -0.123), -1.62 (-3.17 to -0.0729), and -1.44 (-2.48 to -0.403), respectively. The remaining interventions did not differ significantly from placebo. Pairwise comparisons between interventions are shown in **Table 3**.

**Table 3:** League table of network meta-analysis results for intermediate-term pain relief (4–12 weeks).

Intervention	ASTM	Band	Brace	BT	CSI	CT	DPT	ESWT	EX	GAGPS	IONTO	KT	LA	MET	Placebo	PP	PRP	PT	PU	Splint	US
ASTM	ASTM	3.43 (-5.53, 12.17)	3.11 (-5.64, 11.73)	2.98 (-5.78, 11.6)	2.94 (-5.68, 11.48)	3.62 (-5.61, 12.54)	3.82 (-4.85, 12.28)	3.41 (-5.24, 11.91)	3.21 (-5.68, 11.87)	-5.35 (-17.9, 6.17)	3.72 (-5.18, 12.43)	1.97 (-6.67, 10.5)	3.38 (-5.31, 11.9)	4.36 (-4.59, 13.13)	4.56 (-4.03, 13.01)	3.94 (-4.93, 12.65)	4.24 (-4.45, 12.81)	3.12 (-5.58, 11.65)	2.93 (-5.78, 11.49)	3.53 (-5.29, 12.11)	3.1 (-5.5, 11.61)
Band	-3.43 (-12.17, 5.53)	Band	-0.29 (-3.19, 2.56)	-0.44 (-3.48, 2.59)	-0.45 (-3.12, 2.17)	0.19 (-3.58, 3.91)	0.39 (-2.37, 3.12)	0 (-2.27, 2.26)	-0.21 (-3.12, 2.65)	-8.91 (-17.86, -0.24)	0.31 (-2.93, 3.46)	-1.43 (-4.19, 1.18)	-0.04 (-2.77, 2.65)	0.94 (-2.6, 4.39)	1.14 (-1.4, 3.68)	0.53 (-2.76, 3.73)	0.84 (-2.1, 3.67)	-0.3 (-3.01, 2.41)	-0.47 (-3.4, 2.41)	0.11 (-1.53, 1.73)	-0.3 (-2.94, 2.31)
Brace	-3.11 (-11.73, 5.64)	0.29 (-2.56, 3.19)	Brace	-0.14 (-2.45, 2.18)	-0.17 (-2.07, 1.76)	0.47 (-2.97, 3.95)	0.68 (-1.2, 2.61)	0.29 (-1.46, 2.08)	0.08 (-2.43, 2.6)	-8.61 (-17.37, -0.22)	0.59 (-1.68, 2.86)	-1.15 (-3.15, 0.81)	0.25 (-1.23, 1.74)	1.23 (-1.78, 4.25)	1.43 (-0.12, 3.02)	0.81 (-1.49, 3.14)	1.13 (-0.98, 3.18)	-0.01 (-2, 2.02)	-0.18 (-2.37, 2.01)	0.39 (-1.96, 2.77)	-0.02 (-1.84, 1.84)
BT	-2.98 (-11.6, 5.78)	0.44 (-2.59, 3.48)	0.14 (-2.18, 2.45)	BT	-0.03 (-2.08, 2.03)	0.62 (-2.97, 4.2)	0.82 (-1.17, 2.82)	0.44 (-1.57, 2.46)	0.22 (-2.47, 2.89)	-8.47 (-17.23, -0.04)	0.73 (-2.04, 3.48)	-1 (-3.16, 1.08)	0.39 (-1.8, 2.55)	1.37 (-1.73, 4.46)	1.57 (-0.11, 3.28)	0.96 (-1.85, 3.75)	1.27 (-0.94, 3.43)	0.13 (-2.01, 2.28)	-0.05 (-2.33, 2.26)	0.54 (-2.04, 3.1)	0.13 (-1.85, 2.12)
CSI	-2.94 (-11.48, 5.68)	0.45 (-2.17, 3.12)	0.17 (-1.76, 2.07)	0.03 (-2.03, 2.08)	CSI	0.64 (-2.61, 3.92)	0.84 (-0.73, 2.44)	0.47 (-0.88, 1.81)	0.24 (-1.97, 2.47)	-8.44 (-17.12, -0.11)	0.76 (-1.68, 3.17)	-0.98 (-2.32, 0.29)	0.42 (-1.3, 2.12)	1.4 (-0.94, 3.71)	1.6 (0.44, 2.77)	0.98 (-1.5, 3.44)	1.29 (-0.48, 3.04)	0.16 (-1.12, 1.45)	-0.02 (-1.88, 1.84)	0.56 (-1.51, 2.63)	0.15 (-1.23, 1.57)
CT	-3.62 (-12.54, 5.61)	-0.19 (-3.91, 3.58)	-0.47 (-3.95, 2.97)	-0.62 (-4.2, 2.97)	-0.64 (-3.92, 2.61)	CT	0.21 (-3.13, 3.55)	-0.18 (-3.15, 2.79)	-0.4 (-2.78, 1.98)	-9.09 (-18.27, -0.2)	0.12 (-3.63, 3.82)	-1.62 (-4.99, 1.63)	-0.22 (-3.59, 3.07)	0.75 (-3.24, 4.77)	0.96 (-2.22, 4.13)	0.34 (-3.41, 4.09)	0.65 (-2.83, 4.07)	-0.48 (-3.82, 2.82)	-0.67 (-4.14, 2.8)	-0.08 (-3.42, 3.29)	-0.49 (-3.74, 2.74)
DPT	-3.82 (-12.28, 4.85)	-0.39 (-3.12, 2.37)	-0.68 (-2.61, 1.2)	-0.82 (-2.82, 1.17)	-0.84 (-2.44, 0.73)	-0.21 (-3.55, 3.13)	DPT	-0.38 (-1.91, 1.14)	-0.61 (-2.94, 1.73)	-9.28 (-17.98, -1.01)	-0.09 (-2.54, 2.34)	-1.83 (-3.54, -0.21)	-0.42 (-2.18, 1.29)	0.54 (-2.27, 3.34)	0.75 (-0.31, 1.82)	0.14 (-2.36, 2.61)	0.45 (-1.32, 2.16)	-0.69 (-2.4, 1.01)	-0.87 (-2.76, 0.99)	-0.29 (-2.48, 1.89)	-0.7 (-2.18, 0.8)
ESWT	-3.41 (-11.91, 5.24)	0 (-2.26, 2.27)	-0.29 (-2.08, 1.46)	-0.44 (-2.46, 1.57)	-0.47 (-1.81, 0.88)	0.18 (-2.79, 3.15)	0.38 (-1.14, 1.91)	ESWT	-0.22 (-2.01, 1.56)	-8.91 (-17.56, -0.55)	0.3 (-1.98, 2.53)	-1.44 (-2.9, -0.07)	-0.04 (-1.53, 1.41)	0.93 (-1.77, 3.59)	1.14 (0.04, 2.24)	0.53 (-1.8, 2.82)	0.83 (-0.96, 2.57)	-0.31 (-1.78, 1.19)	-0.49 (-2.28, 1.32)	0.1 (-1.47, 1.67)	-0.31 (-1.59, 0.98)
EX	-3.21 (-11.87, 5.68)	0.21 (-2.65, 3.12)	-0.08 (-2.6, 2.43)	-0.22 (-2.89, 2.47)	-0.24 (-2.47, 1.97)	0.4 (-1.98, 2.78)	0.61 (-1.73, 2.94)	0.22 (-1.56, 2.01)	EX	-8.68 (-17.55, -0.15)	0.52 (-2.37, 3.38)	-1.22 (-3.53, 1.02)	0.18 (-2.15, 2.48)	1.15 (-2.07, 4.37)	1.36 (-0.72, 3.45)	0.75 (-2.19, 3.67)	1.05 (-1.46, 3.52)	-0.08 (-2.4, 2.21)	-0.26 (-2.79, 2.25)	0.32 (-2.03, 2.69)	-0.09 (-2.28, 2.11)
GAGPS	5.35 (-6.17, 17.9)	8.91 (0.24, 17.86)	8.61 (0.22, 17.37)	8.47 (0.04, 17.23)	8.44 (0.11, 17.12)	9.09 (0.2, 18.27)	9.28 (1.01, 17.98)	8.91 (0.55, 17.56)	8.68 (0.15, 17.55)	GAGPS	9.21 (0.66, 18.04)	7.45 (-0.84, 16.14)	8.86 (0.49, 17.53)	9.84 (1.16, 18.82)	10.04 (1.81, 18.64)	9.42 (0.86, 18.3)	9.73 (1.42, 18.46)	8.61 (0.22, 17.31)	8.43 (0.03, 17.17)	9.01 (0.5, 17.77)	8.6 (0.3, 17.28)
IONTO	-3.72 (-12.43, 5.18)	-0.31 (-3.46, 2.93)	-0.59 (-2.86, 1.68)	-0.73 (-3.48, 2.04)	-0.76 (-3.17, 1.68)	-0.12 (-3.82, 3.63)	0.09 (-2.34, 2.54)	-0.3 (-2.53, 1.98)	-0.52 (-3.38, 2.37)	-9.21 (-18.04, -0.66)	IONTO	-1.74 (-4.24, 0.7)	-0.34 (-2.06, 1.35)	0.63 (-2.72, 4)	0.84 (-1.33, 3.04)	0.22 (-1.5, 1.95)	0.54 (-2.08, 3.09)	-0.6 (-3.09, 1.92)	-0.78 (-3.43, 1.89)	-0.2 (-2.93, 2.55)	-0.61 (-2.95, 1.77)
KT	-1.97 (-10.5, 6.67)	1.43 (-1.18, 4.19)	1.15 (-0.81, 3.15)	1 (-1.08, 3.16)	0.98 (-0.29, 2.32)	1.62 (-1.63, 4.99)	1.83 (0.21, 3.54)	1.44 (0.07, 2.9)	1.22 (-1.02, 3.53)	-7.45 (-16.14, 0.84)	1.74 (-0.7, 4.24)	KT	1.39 (-0.35, 3.22)	2.37 (-0.25, 5.06)	2.58 (1.32, 3.92)	1.97 (-0.54, 4.52)	2.27 (0.43, 4.15)	1.14 (-0.45, 2.81)	0.96 (-0.87, 2.88)	1.53 (-0.53, 3.71)	1.13 (-0.12, 2.5)
LA	-3.38 (-11.9, 5.31)	0.04 (-2.65, 2.77)	-0.25 (-1.74, 1.23)	-0.39 (-2.55, 1.8)	-0.42 (-2.12, 1.3)	0.22 (-3.07, 3.59)	0.42 (-1.29, 2.18)	0.04 (-1.41, 1.53)	-0.18 (-2.48, 2.15)	-8.86 (-17.53, -0.49)	0.34 (-1.35, 2.06)	-1.39 (-3.22, 0.35)	LA	0.98 (-1.9, 3.86)	1.18 (-0.17, 2.56)	0.57 (-1.2, 2.34)	0.88 (-1.08, 2.8)	-0.26 (-2.07, 1.58)	-0.44 (-2.45, 1.61)	0.14 (-2, 2.31)	-0.26 (-1.89, 1.39)
MET	-4.36 (-13.13, 4.59)	-0.94 (-4.39, 2.6)	-1.23 (-4.25, 1.78)	-1.37 (-4.46, 1.73)	-1.4 (-3.71, 0.94)	-0.75 (-4.77, 3.24)	-0.54 (-3.34, 2.27)	-0.93 (-3.59, 1.77)	-1.15 (-4.37, 2.07)	-9.84 (-18.82, -1.16)	-0.63 (-4, 2.72)	-2.37 (-5.06, 0.25)	-0.98 (-3.86, 1.9)	MET	0.2 (-2.38, 2.81)	-0.42 (-3.81, 2.97)	-0.11 (-3.03, 2.78)	-1.24 (-3.86, 1.43)	-1.42 (-4.36, 1.58)	-0.83 (-3.93, 2.3)	-1.24 (-3.95, 1.48)
Placebo	-4.56 (-13.01, 4.03)	-1.14 (-3.68, 1.4)	-1.43 (-3.02, 0.12)	-1.57 (-3.28, 0.11)	-1.6 (-2.77, -0.44)	-0.96 (-4.13, 2.22)	-0.75 (-1.82, 0.31)	-1.14 (-2.24, -0.04)	-1.36 (-3.45, 0.72)	-10.04 (-18.64, -1.81)	-0.84 (-3.04, 1.33)	-2.58 (-3.92, -1.32)	-1.18 (-2.56, 0.17)	-0.2 (-2.81, 2.38)	Placebo	-0.62 (-2.87, 1.62)	-0.3 (-1.71, 1.06)	-1.44 (-2.77, -0.12)	-1.62 (-3.17, -0.07)	-1.04 (-2.97, 0.88)	-1.44 (-2.48, -0.4)
PP	-3.94 (-12.65, 4.93)	-0.53 (-3.73, 2.76)	-0.81 (-3.14, 1.49)	-0.96 (-3.75, 1.85)	-0.98 (-3.44, 1.5)	-0.34 (-4.09, 3.41)	-0.14 (-2.61, 2.36)	-0.53 (-2.82, 1.8)	-0.75 (-3.67, 2.19)	-9.42 (-18.3, -0.86)	-0.22 (-1.95, 1.5)	-1.97 (-4.52, 0.54)	-0.57 (-2.34, 1.2)	0.42 (-2.97, 3.81)	0.62 (-1.62, 2.87)	PP	0.31 (-2.37, 2.93)	-0.83 (-3.36, 1.74)	-1 (-3.71, 1.7)	-0.42 (-3.19, 2.38)	-0.83 (-3.23, 1.6)
PRP	-4.24 (-12.81, 4.45)	-0.84 (-3.67, 2.1)	-1.13 (-3.18, 0.98)	-1.27 (-3.43, 0.94)	-1.29 (-3.04, 0.48)	-0.65 (-4.07, 2.83)	-0.45 (-2.16, 1.32)	-0.83 (-2.57, 0.96)	-1.05 (-3.52, 1.46)	-9.73 (-18.46, -1.42)	-0.54 (-3.09, 2.08)	-2.27 (-4.15, -0.43)	-0.88 (-2.8, 1.08)	0.11 (-2.78, 3.03)	0.3 (-1.06, 1.71)	-0.31 (-2.93, 2.37)	PRP	-1.13 (-2.99, 0.76)	-1.31 (-3.35, 0.76)	-0.73 (-3.07, 1.67)	-1.14 (-2.83, 0.61)
PT	-3.12 (-11.65, 5.58)	0.3 (-2.41, 3.01)	0.01 (-2.02, 2)	-0.13 (-2.28, 2.01)	-0.16 (-1.45, 1.12)	0.48 (-2.82, 3.82)	0.69 (-1.01, 2.4)	0.31 (-1.19, 1.78)	0.08 (-2.21, 2.4)	-8.61 (-17.31, -0.22)	0.6 (-1.92, 3.09)	-1.14 (-2.81, 0.45)	0.26 (-1.58, 2.07)	1.24 (-1.43, 3.86)	1.44 (0.12, 2.77)	0.83 (-1.74, 3.36)	1.13 (-0.76, 2.99)	PT	-0.18 (-2.15, 1.8)	0.41 (-1.76, 2.56)	0 (-1.58, 1.59)
PU	-2.93 (-11.49, 5.78)	0.47 (-2.41, 3.4)	0.18 (-2.01, 2.37)	0.05 (-2.26, 2.33)	0.02 (-1.84, 1.88)	0.67 (-2.8, 4.14)	0.87 (-0.99, 2.76)	0.49 (-1.32, 2.28)	0.26 (-2.25, 2.79)	-8.43 (-17.17, -0.03)	0.78 (-1.89, 3.43)	-0.96 (-2.88, 0.87)	0.44 (-1.61, 2.45)	1.42 (-1.58, 4.36)	1.62 (0.07, 3.17)	1 (-1.7, 3.71)	1.31 (-0.76, 3.35)	0.18 (-1.8, 2.15)	PU	0.58 (-1.8, 2.97)	0.17 (-1.34, 1.71)
Splint	-3.53 (-12.11, 5.29)	-0.11 (-1.73, 1.53)	-0.39 (-2.77, 1.96)	-0.54 (-3.1, 2.04)	-0.56 (-2.63, 1.51)	0.08 (-3.29, 3.42)	0.29 (-1.89, 2.48)	-0.1 (-1.67, 1.47)	-0.32 (-2.69, 2.03)	-9.01 (-17.77, -0.5)	0.2 (-2.55, 2.93)	-1.53 (-3.71, 0.53)	-0.14 (-2.31, 2)	0.83 (-2.3, 3.93)	1.04 (-0.88, 2.97)	0.42 (-2.38, 3.19)	0.73 (-1.67, 3.07)	-0.41 (-2.56, 1.76)	-0.58 (-2.97, 1.8)	Splint	-0.41 (-2.42, 1.62)
US	-3.1 (-11.61, 5.5)	0.3 (-2.31, 2.94)	0.02 (-1.84, 1.84)	-0.13 (-2.12, 1.85)	-0.15 (-1.57, 1.23)	0.49 (-2.74, 3.74)	0.7 (-0.8, 2.18)	0.31 (-0.98, 1.59)	0.09 (-2.11, 2.28)	-8.6 (-17.28, -0.3)	0.61 (-1.77, 2.95)	-1.13 (-2.5, 0.12)	0.26 (-1.39, 1.89)	1.24 (-1.48, 3.95)	1.44 (0.4, 2.48)	0.83 (-1.6, 3.23)	1.14 (-0.61, 2.83)	0 (-1.59, 1.58)	-0.17 (-1.71, 1.34)	0.41 (-1.62, 2.42)	US

Results are presented as mean differences (MDs) with 95% credible intervals (CrIs) for pairwise comparisons between interventions. All pain outcomes were standardized to a 0–10 visual analog scale (VAS). A negative MD indicates that the treatment listed in the row was associated with greater pain reduction than the treatment listed in the column. Comparisons with 95%CrIs not crossing 0 were considered statistically significant.

**Figure 9:**
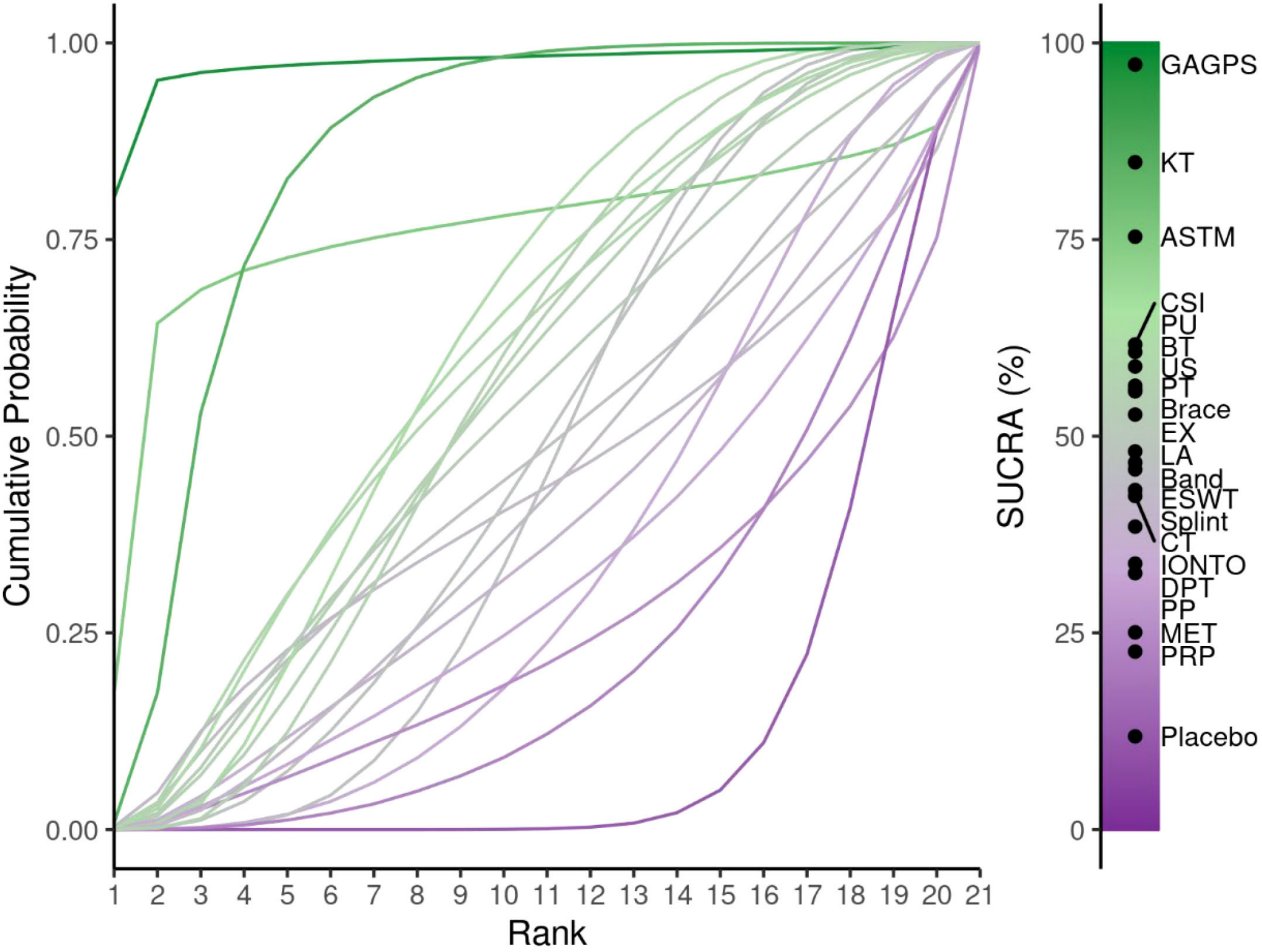
Cumulative ranking probability curves and SUCRA values for intermediate-term pain relief (4-12 weeks). The cumulative ranking curves illustrate the probability of each intervention occupying each possible rank for intermediate term pain relief. SUCRA values summarize the overall ranking performance of each treatment, with higher values indicating a greater likelihood of better pain-relieving efficacy.

The cumulative ranking probability curves are shown in **Figure 9**, and ranking probabilities and SUCRA values are provided in **Supplementary Table 3**. GAGPS, KT, and ASTM ranked relatively high for intermediate-term pain relief, with SUCRA values of 97.24%, 84.83%, and 75.36%, respectively. Placebo ranked lowest (SUCRA = 11.85%). However, although ASTM ranked relatively high, its 95%CrI versus placebo crossed the line of no effect; therefore, ranking results should be interpreted cautiously.

**Figure 10:**
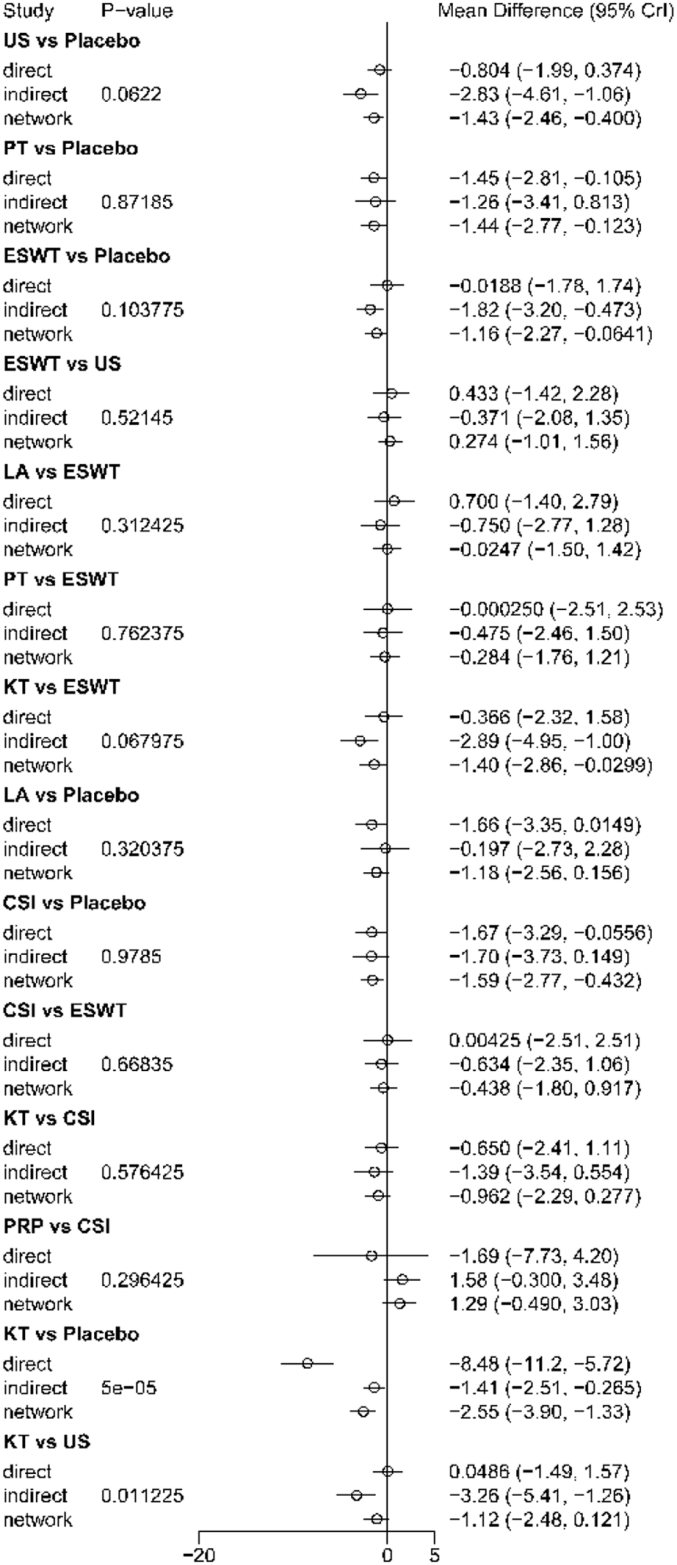
Node-splitting analysis for intermediate-term outcomes (4-12 weeks). The figure presents the results of the node-splitting analysis used to assess local inconsistency between direct and indirect evidence in the intermediate-term network. Comparisons with non-significant differences indicate no evidence of important inconsistency between direct and indirect estimates.

Node-splitting results are shown in **Figure 10**. Most comparisons showed no significant inconsistency, but significant local inconsistency was observed for KT versus placebo and KT versus US, indicating differences between direct and indirect evidence for some comparisons in the intermediate-term network.

### Long-term outcomes

**Figure 11:**
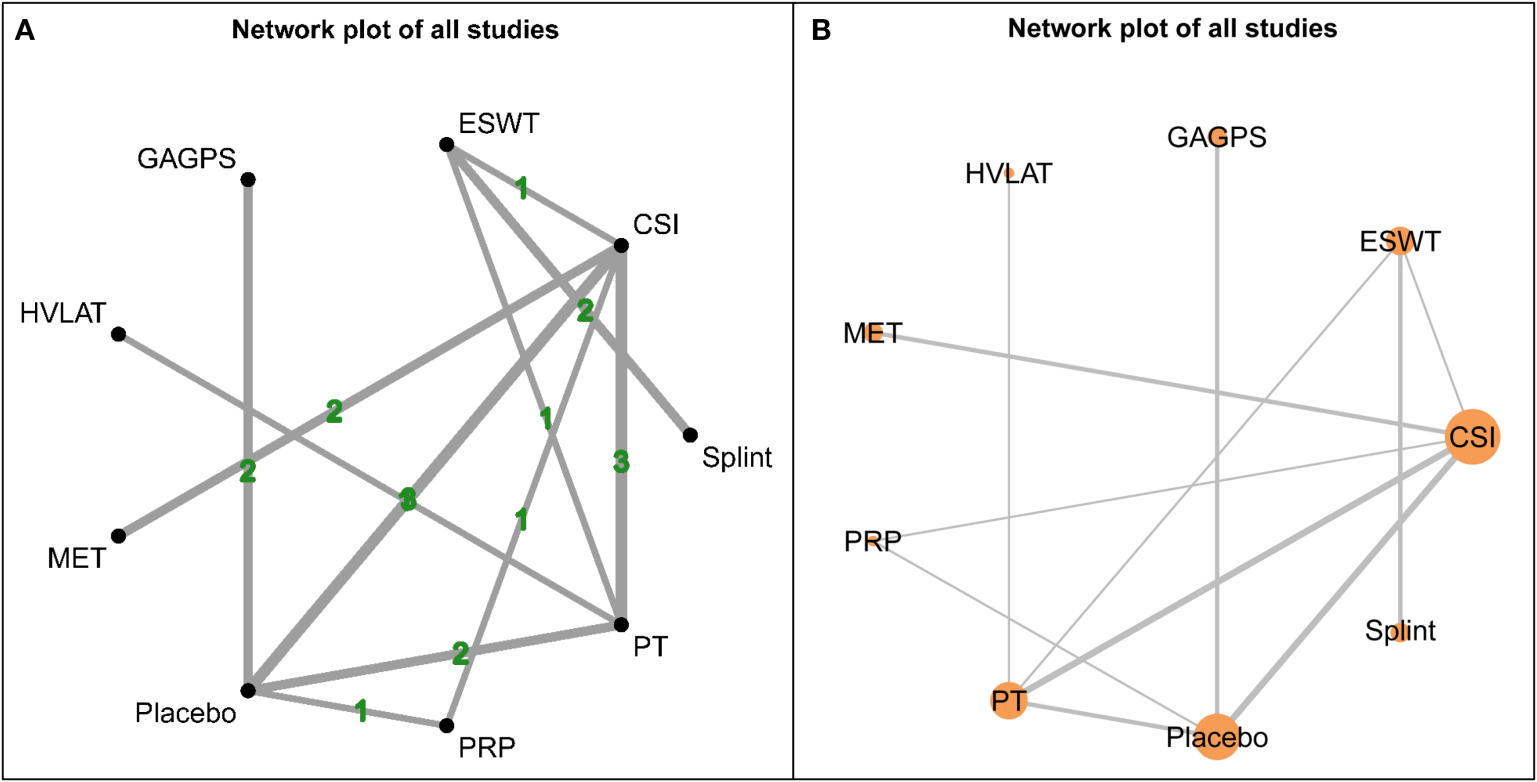
Network plots for long-term outcomes (>12 weeks). **(A)** shows the network structure weighted by the number of direct comparisons, with edge thickness proportional to the number of studies contributing to each comparison. **(B)** shows the network structure weighted by the number of participants assigned to each intervention, with node size proportional to sample size.

The network structure for long-term outcomes is shown in **Figures 11A, B**. Nine interventions were included in this time window: CSI, ESWT, GAGPS, HVLAT, MET, placebo, PRP, PT, and splint. Compared with the short- and intermediate-term networks, the long-term network was considerably sparser, and placebo, CSI, and PT served as the major connecting nodes. Baseline characteristics relevant to transitivity assessment are presented in **Supplementary Table 1**.

**Figure 12:**
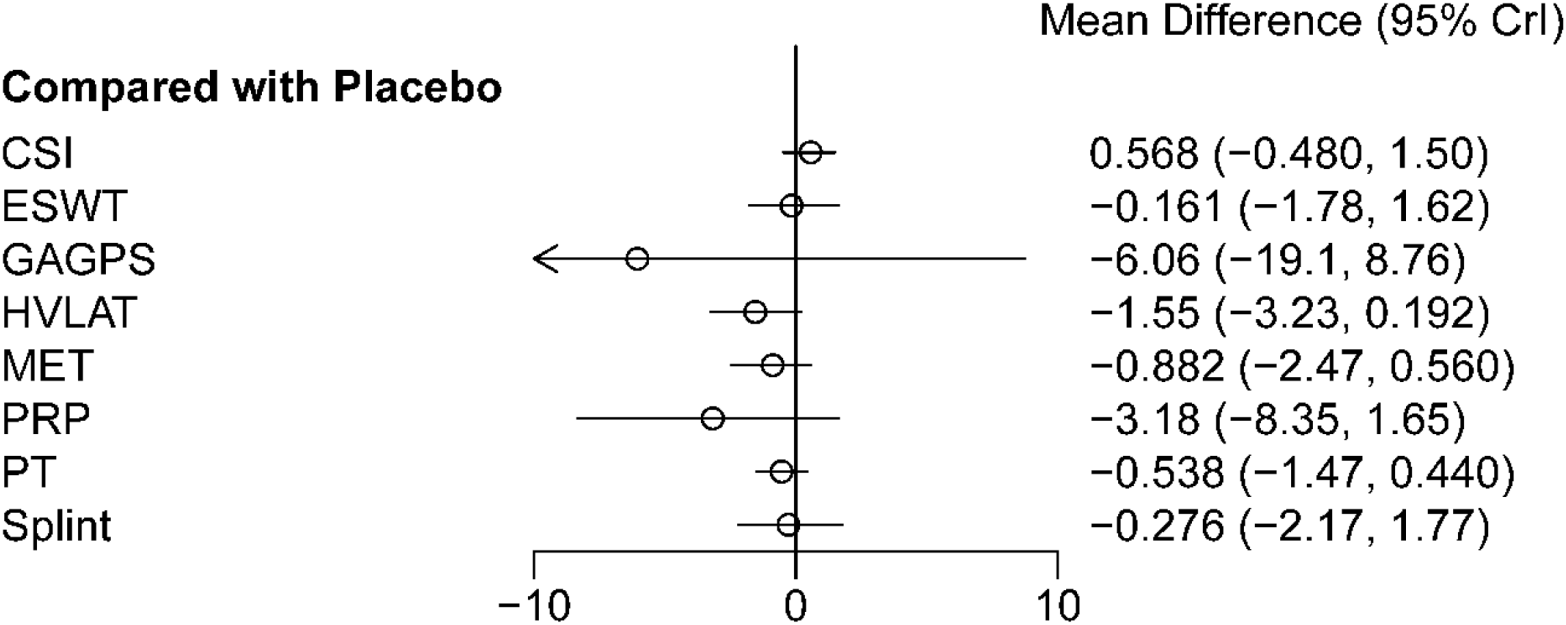
Forest plot of long-term treatment effects on pain relief (>12 weeks). The figure presents the relative effects of each intervention compared with placebo for long-term pain relief, expressed as mean differences (MDs) with 95% credible intervals (CrIs). Negative MD values indicate greater pain reduction and therefore a more favorable treatment effect.

Forest plot results (**Figure 12**) showed that none of the interventions demonstrated a statistically significant advantage over placebo for long-term pain relief. The MDs (95%CrIs) versus placebo were 0.568 (-0.480 to 1.50) for CSI, -0.161 (-1.78 to 1.62) for ESWT, -6.06 (-19.1 to 8.76) for GAGPS, -1.55 (-3.23 to 0.192) for HVLAT, -0.882 (-2.47 to 0.560) for MET, -3.18 (-8.35 to 1.65) for PRP, -0.538 (-1.47 to 0.440) for PT, and -0.276 (-2.17 to 1.77) for splint. Pairwise comparisons between interventions are shown in **Table 4**.

**Table 4:** League table of network meta-analysis results for long-term pain relief (>12 weeks).

Intervention	CSI	ESWT	GAGPS	HVLAT	MET	Placebo	PRP	PT	Splint
CSI	CSI	-0.73 (-2.16, 0.93)	-6.61 (-19.69, 8.21)	-2.12 (-3.71, -0.37)	-1.45 (-2.63, -0.32)	-0.57 (-1.5, 0.48)	-3.73 (-8.92, 1.1)	-1.11 (-1.89, -0.16)	-0.84 (-2.56, 1.11)
ESWT	0.73 (-0.93, 2.16)	ESWT	-5.92 (-19.04, 8.94)	-1.38 (-3.51, 0.59)	-0.73 (-2.78, 1.05)	0.16 (-1.62, 1.78)	-3.04 (-8.44, 2)	-0.38 (-1.92, 1.08)	-0.11 (-1.12, 0.88)
GAGPS	6.61 (-8.21, 19.69)	5.92 (-8.94, 19.04)	GAGPS	4.54 (-10.33, 17.65)	5.16 (-9.67, 18.17)	6.06 (-8.76, 19.12)	2.79 (-12.61, 17.14)	5.53 (-9.26, 18.59)	5.81 (-9.13, 18.95)
HVLAT	2.12 (0.37, 3.71)	1.38 (-0.59, 3.51)	-4.54 (-17.65, 10.33)	HVLAT	0.66 (-1.45, 2.6)	1.55 (-0.19, 3.23)	-1.61 (-6.99, 3.41)	1 (-0.39, 2.44)	1.27 (-0.96, 3.61)
MET	1.45 (0.32, 2.63)	0.73 (-1.05, 2.78)	-5.16 (-18.17, 9.67)	-0.66 (-2.6, 1.45)	MET	0.88 (-0.56, 2.47)	-2.29 (-7.62, 2.7)	0.34 (-1.02, 1.88)	0.62 (-1.42, 2.91)
Placebo	0.57 (-0.48, 1.5)	-0.16 (-1.78, 1.62)	-6.06 (-19.12, 8.76)	-1.55 (-3.23, 0.19)	-0.88 (-2.47, 0.56)	Placebo	-3.18 (-8.35, 1.65)	-0.54 (-1.47, 0.44)	-0.28 (-2.17, 1.77)
PRP	3.73 (-1.1, 8.92)	3.04 (-2, 8.44)	-2.79 (-17.14, 12.61)	1.61 (-3.41, 6.99)	2.29 (-2.7, 7.62)	3.18 (-1.65, 8.35)	PRP	2.65 (-2.2, 7.86)	2.91 (-2.18, 8.42)
PT	1.11 (0.16, 1.89)	0.38 (-1.08, 1.92)	-5.53 (-18.59, 9.26)	-1 (-2.44, 0.39)	-0.34 (-1.88, 1.02)	0.54 (-0.44, 1.47)	-2.65 (-7.86, 2.2)	PT	0.27 (-1.51, 2.11)
Splint	0.84 (-1.11, 2.56)	0.11 (-0.88, 1.12)	-5.81 (-18.95, 9.13)	-1.27 (-3.61, 0.96)	-0.62 (-2.91, 1.42)	0.28 (-1.77, 2.17)	-2.91 (-8.42, 2.18)	-0.27 (-2.11, 1.51)	Splint

Results are presented as mean differences (MDs)with 95% credible intervals (CrIs) for pairwise comparisons between interventions. All pain outcomes were standardized to a 0–10 visual analog scale (VAS). A negative MD indicates that the treatment listed in the row was associated with greater pain reduction than the treatment listed in the column. Comparisons with 95%CrIs not crossing 0 were considered statistically significant.

**Figure 13:**
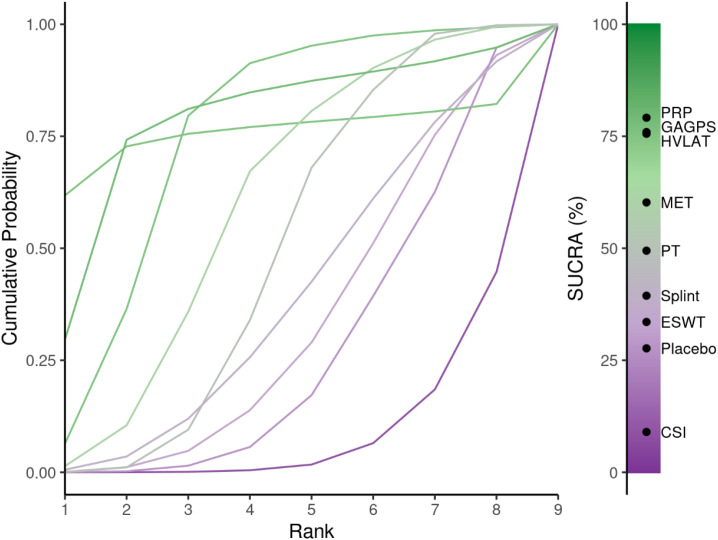
Cumulative ranking probability curves and SUCRA values for long-term pain relief (>12 weeks). The cumulative ranking curves illustrate the probability of each intervention occupying each possible rank for long-term pain relief. SUCRA values summarize the overall ranking performance of each treatment, with higher values indicating a greater likelihood of better pain-relieving efficacy.

The cumulative ranking probability curves are shown in **Figure 13**, and ranking probabilities and SUCRA values are reported in **Supplementary Table 4**. PRP, GAGPS, and HVLAT ranked relatively high for long-term pain relief, with SUCRA values of 79.14%, 75.93%, and 75.56%, respectively, whereas CSI ranked lowest (SUCRA = 9.01%). However, although PRP, GAGPS, and HVLAT ranked relatively high, their 95%CrIs versus placebo crossed the line of no effect, and several comparisons had wide credible intervals; therefore, long-term ranking results should be interpreted cautiously.

**Figure 14:**
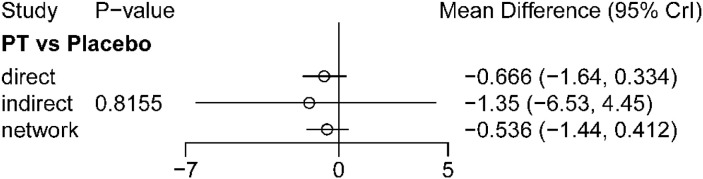
Node-splitting analysis for long-term outcomes (>12 weeks). The figure presents the results of the node-splitting analysis used to assess local inconsistency between direct and indirect evidence in the long-term network. Comparisons with non-significant differences indicate no evidence of important inconsistency between direct and indirect estimates.

Node-splitting results are shown in **Figure 14**. Only PT versus placebo could be tested using node-splitting, and no significant local inconsistency was detected (P = 0.8155), suggesting overall agreement between direct and indirect evidence for the testable comparison in the long-term network.

## Discussion

This updated Bayesian network meta-analysis compared the time-dependent pain-relieving efficacy of multiple non-surgical interventions for lateral epicondylitis. Overall, the analgesic effects of different interventions varied across follow-up stages: some interventions appeared relatively favorable in the short and intermediate term, whereas no clear long-term superiority was observed. This general pattern is broadly consistent with recent summaries of the evidence on LE and tendinopathy, which suggest that pain improvement is more readily observed in the short and intermediate term, whereas long-term superiority is often limited by evidence quality, study number, and network sparsity (Hardy et al., 2021; Lapner et al., 2022; Hohmann et al., 2023; Karbowiak et al., 2023; Benzon et al., 2025). Our findings also reinforce that treatment rankings provide only relative information and should be interpreted together with effect estimates, interval width, direct evidence, and consistency diagnostics rather than on the basis of SUCRA alone (Benzon et al., 2025).

Some of our findings were consistent with previous studies. Corticosteroid injection generally provides rapid short-term pain relief, whereas its intermediate- and long-term advantages are limited; this agrees with recent systematic reviews and meta-analyses comparing PRP with corticosteroids (Hardy et al., 2021; Hohmann et al., 2023). PRP, in contrast, appears more likely to confer benefit in the medium to long term rather than in the early phase, which is also consistent with recent LE-specific evidence syntheses (Chen et al., 2021; Hardy et al., 2021). ESWT showed a relative advantage in the intermediate-term results of the present study, which is in line with prior network meta-analyses suggesting competitive performance of ESWT for pain relief (Hohmann et al., 2023; Benzon et al., 2025). In addition, the relatively favorable intermediate-term result for PT is consistent with recent tendinopathy rehabilitation literature emphasizing the value of load management and exercise-based treatment (Pavlova et al., 2023).

At the same time, our results were not entirely consistent with all previous studies. One likely explanation is the inclusion of newly identified randomized controlled trials after the updated search, which changed the network structure, the number of direct comparisons, and the relative effect estimates. In addition, all pain outcomes in the present study were converted to a common 0–10 VAS scale, and results were reported as MDs with 95%CrIs within a Bayesian framework; this differs from some previous studies that used SMDs, did not standardize pain scales, or did not strictly separate follow-up stages. Furthermore, the LE literature is characterized by complex intervention classifications, heterogeneous comparator definitions, and variable follow-up schedules, making some divergence between systematic reviews methodologically unsurprising (Campos et al., 2024). Therefore, discrepancies between our findings and earlier studies are more likely to reflect differences in updated evidence, analytical framework, and time-window handling than mutually exclusive conclusions.

The absence of clear statistically significant superiority in the long term is one of the most important findings of this study. First, this result should be interpreted in light of the evidence structure: compared with the short- and intermediate-term networks, the long-term network included fewer studies, fewer intervention nodes, and fewer direct comparisons, making long-term relative effect estimates more uncertain (Lapner et al., 2022; Kim et al., 2024; Benzon et al., 2025). Second, the interventions entering the long-term analysis were limited in scope and did not fully represent all therapies that performed relatively well in the short and intermediate term; therefore, the long-term network reflects only interventions currently supported by long-term follow-up evidence rather than the full long-term potential of all non-surgical treatments (Lapner et al., 2022; Kim et al., 2024). From the perspective of disease course, LE belongs to the spectrum of tendinopathy, and persistence of long-term symptoms may depend not only on local tissue status but also on sustained mechanical loading, tissue adaptation, behavioral adjustment, and pain modulation (Karanasios et al., 2021; Millar et al., 2021; Pavlova et al., 2023). Consequently, an intervention that shows analgesic benefit in the short or intermediate term may not naturally maintain that advantage over the long term.

Considering the interventions included in the long-term analysis, namely CSI, ESWT, GAGPS, HVLAT, MET, PRP, PT, and splint, their therapeutic targets are not identical. Some interventions are more oriented toward short-term pain modulation, whereas others may depend more heavily on ongoing exercise progression, load management, or staged rehabilitation to sustain benefit. For example, exercise-based treatments in tendinopathy may not function primarily as immediate analgesics, but rather through gradual load exposure, tissue adaptation, and restoration of function; their effects are therefore more likely to depend on dose, progression criteria, and adherence (Karanasios et al., 2021; Pavlova et al., 2023). By contrast, injection-based treatments may produce more rapid symptom relief, but their long-term effects are more easily influenced by biological tissue responses and the natural history of the condition (Hardy et al., 2021; Hohmann et al., 2023). Accordingly, the lack of long-term statistical superiority is more reasonably interpreted as evidence insufficient to support a clear long-term superior intervention, rather than proof that all non-surgical interventions are ineffective in the long term.

From a mechanistic perspective, only cautious interpretive inferences can be made. The short-term advantage of KT and brace may be related to improved load distribution, altered sensory input, and changes in pain perception (Millar et al., 2021). The short- to intermediate-term effect of CSI is compatible with its rapid anti-inflammatory and analgesic action, although prior evidence suggests that its long-term value should be interpreted cautiously (Dean et al., 2014). The relative intermediate-term advantage of ESWT may indicate that its effect is not limited to immediate analgesia, but may also be related to local tissue adaptation, pain modulation, and functional recovery processes (Liu et al., 2022). However, tendinopathy research has also shown that pain improvement and tissue status are not simply linearly related, and chronic symptoms may be influenced by interacting mechanical, biological, and neural regulatory factors (Millar et al., 2021). Thus, the mechanistic interpretations offered here should be regarded as evidence-based hypotheses rather than causal mechanisms directly demonstrated by the present study.

Clinically, our findings support a stage-specific and dynamic approach to non-surgical management of LE. For patients whose primary goal is short-term pain reduction, the short- and intermediate-term results may help inform treatment selection. However, for patients with prolonged, recurrent, or persistent symptoms, treatment decisions should not be based solely on rankings at a single time point, but should also take into account disease stage, treatment goals, patient adherence, and the sufficiency of direct evidence. This further suggests that a more clinically meaningful future direction may not be to identify one treatment that is best across all stages, but rather to explore more rational sequential, combined, or individualized treatment strategies for different stages of the condition.

Several limitations should be acknowledged. First, this study focused primarily on pain outcomes measured by the VAS and therefore cannot fully reflect broader clinical value, such as functional recovery, grip strength, return to work or sport, and recurrence; this limitation has also been noted in recent LE evidence syntheses (Benzon et al., 2025). Second, some included studies provided insufficient information on baseline characteristics, symptom duration, and follow-up details, limiting deeper evaluation of transitivity and potential effect modifiers (Kim et al., 2021). Third, the long-term network was sparse, and some findings were based on limited direct evidence, leading to greater uncertainty in long-term conclusions. Future studies should prioritize long-term randomized controlled trials, direct head-to-head comparisons between active interventions, and the inclusion of functional, grip-strength, and patient-reported outcomes to provide a more comprehensive assessment of the true clinical value of stage-specific non-surgical treatment strategies.

## Conclusion

This updated Bayesian network meta-analysis showed that the analgesic effects of non-surgical interventions for lateral epicondylitis are time-dependent. Some treatments demonstrated relative advantages in the short and intermediate term, but no intervention showed a clear long-term advantage over placebo. These findings suggest that treatment efficacy should be interpreted in the context of follow-up stage, effect estimates, and evidence stability rather than treatment ranking alone. Further high-quality randomized controlled trials with longer follow-up are needed to clarify the long-term value of different non-surgical strategies.
