# Supplementary material for: Time-dependent comparative efficacy of non-surgical treatments for pain relief in lateral epicondylitis: a systematic review and network meta-analysis
Source: Front Physiol. 2026 Mar 27;17:1782562. doi: 10.3389/fphys.2026.1782562 (PMC13066320; doi:10.3389/fphys.2026.1782562)
Supplement: Supplementary file 1 [file DataSheet1.docx]

Figures and tables

Figure 1．Flow diagram of study selection. The figure illustrates the identification, screening, full-text assessment, and final inclusion of studies in the updated review. Based on the 24 studies included in the previous version of the systematic review, 3 additional studies were identified through the updated search, resulting in a total of 27 included studies.

Table 1. Intervention-Related Characteristics of the Included Randomized Controlled Trials

| Study | Diagnosis reported | Duration (month) | Sample size (F/M) | Mean age (years) | Intervention details | Outcome measure | Follow-up (Weeks) | Main result |
| --- | --- | --- | --- | --- | --- | --- | --- | --- |
| Agarwal et al. 2023 | Yes | NR | 10 10 | 30-45 | HVLAT: high velocity low amplitude thrust  Physiotherapy: 3 sessions/week on alternative days for 1 week | VAS | 24 | HVLAT^+^ |
| Agostinucci et al. 2012 | Yes | ＞3  ＞3 | 74 | NR | EX: traditional exercise  Cryo-Max®: three cycles of 20 minutes cold pack | VAS | 6 | Cryo-Max®+ |
| Akcay et al. 2020 | Yes | 9  18 | 23 (18/5)  27 (19/8) | 48.1  46.7 | DPT: 15% dextrose, 1.5 mL Placebo: 1.5 mL, no peppering | VAS | 0, 4, 8, 12 | Saline+ |
| Akermark et al.,1995 | Yes | 3-36  3-30 | 30 (15/15) 30 (14/16) | 46  42 | GAGPS: 50 rng, one/week, five weeks Placebo: 50 rng, one/week, five weeks | VAS | 3, 2, 12, 26 | GAGPS+ |
| Akin et al. 2010 | Yes | 8.7  6.8 | 30 (18/12)  30 (20/10) | 46.7  45.5 | Ultrasound: 15 US, Petas Petson 250 US 2200 for 1 MHz and 1.5 W/cm2 Placebo: nonworking head for 5 minutes | VAS | 3, 5 | US+ |
| Aydin et al. 2018 | Yes | 1  1 | 32  (17/15)  35  (16/19) | 38.84  37.94 | ESWT: 4 sessions per week, 10-12 Hz, 2,000 pulses, and 1.6-1.8 bar pressure  WES: a wrist splint at 30°–45° extension for 4 weeks | VAS | 4, 12, 24 | WES+ |
| Baktir et al. 2019 | Yes | 11  12.25  12 | 12 (10/2)  12 (9/3)  13 (10/3) | 45.33  43.75  49.31 | Laser: 904 nm wavelength of, 57000 Hz, peak power of 27 W, 50 W, or 27×4W Phonophoresis: 2 mg/d prednisolone, 5 cm2 US head, 1 W/cm2 dosage, 1 MHz, 7 minutes Iontophoresis: 5 mL of 0.4% prednisolone | VAS | 3 | Iontophoresis+ |
| Bisset et al. 2006 | Yes | 6.06  6.06  3.7 | 67 (24/43)  65 (25/40)  66 (21/45) | 47.3  47.8  47.9 | WAS: wait and see CI: 1 ml of 1% lidocaine with 10 mg of triaminolone acetonide in 1 ml PT: eight treatments of 30 minutes, 6 weeks | VAS | 3, 6, 12, 26, 52 | CI+ |
| Blanchette et al. 2011 | Yes | 22 43 | 15 (9/6)  12 (6/6) | 47  46 | ASTM: 2 times/week/5 weeks Control: extensors muscles of the wrist (hold 30 seconds, 6 times a day) | VAS | 6, 12 | No Difference |
| Çorum et al. 2021 | Yes | ＞3  ＞3 | 22 (16/6) 19 (14/5) | 49  45 | ESWT: once per week with 1.8 bar pressure, 10 Hz, 2,000 pulses. Exercise: 3 times/week for three weeks | VAS | 4, 12 | No Difference |
| Creuze et al. 2018 | Yes | 17.2  20.2 | 30 (13/17) 30 (14/16) | 47.3  46.7 | BoNT-A: 40 IU of BoNT-A from a 500-IU flask, diluted in 5 mL of saline solution Placebo: 0.4 mL of saline solution | VAS | 12 | BoNT-A+ |
| Devrimsel et al. 2014 | Yes | NR | 30 (22/8)  30 (20/10) | 37.76  40.3 | ESWT: 2000 shock waves, 1.6 bar intensity and 16 Hz, 3 times/3 weeks, a 1-week interval Laser: 10 sessions with 3.6 joule intensity, 500 Hz, and 850 nm wavelength, | VAS | 12 | ESWT+ |
| Dundar et al. 2015 | Yes | 28.7  29.5  27.9 | 30 (17/13)  31 (17/14)  30 (15/15) | 32.6  33.4 33.6 | HILT: 360-1780 mJ/cm2, 120-150 μs, 10.5 W, 10-40 Hz Placebo: shame placebo Brace: lateral counterforce brace | VAS | 4, 12 | HILT+ |
| Gündüz et al. 2012 | Yes | 3  3  3 | 19 (14/5)  20 (12/8)  20 (12/8) | 43.6  45.7  44.9 | PT: US 1 W/cm2, 5 min, friction massage 5 min for ten sessions CI: 20 mg methylprednisolone acetate and 1 ml prilocaine ESWT: pressure 1.4 bar, frequency 4.0 Hz, number 500 for ten sessions | VAS | 4. 12. 24 | ESWT+ |
| Huseyin et al. 2021 | Yes | 3.5  3.7  3.7 | 17 (10/7)  17 (12/5)  17 (11/6) | 45.3  47.2  47.1 | CUS: 1.5 MHz and 1 W/cm2 for 5 min per session PUS: 1.5 MHz and 1 W/cm2 a pulsed mode duty cycle of 1:4 Placebo US: received a sham US | VAS | 6 | CUS+  PUS+ |
| Koçak et al. 2019 | Yes | 4.50  5.07 | 28 (14/14)  28 (18/10) | 43.54  40.96 | SI: 20 mg of methylprednisolone acetate (0.5 mL) and 0.5 mL of prilocaine at 2% KT: tape treatment with a 15%-25% stretch,, no stretching | VAS | 3, 12 | KT+ |
| Küçüksen et al. 2013 | Yes | 5.3  6.1 | 41 (23/18)  41 (22/19) | 46.17  43.78 | MET: forearm pronation and supination in 8 sessions, each with 5 repetitions of 5-second contractions CSI: 1mL of triamcinolone (40mg/mL) and 1mL of lidocaine (10mg/mL) | VAS | 6, 26, 52 | MET+ |
| Lam et al. 2007 | Yes | 3.2  3.3 | 21 (12/9)  18 (11/7) | 46.1 48.9 | LA: 5000 Hz, 2.4 J/cm², 11 sec per point; 0.66 J for 2.4 tender points Placebo: sham irradiation | VAS | 3 | LA+ |
| Montalvan et al. 2016 | Yes | ≤3 | 50 | 35-65 | PRP: 12 ml blood; PRP showed 1.6x platelet enrichment, with 2 ml syringes for PRP and saline. Placebo: 2 ml of 1% lidocaine s.c. | VAS | 12 | No Difference |
| Özmen et al. 2021 | Yes | 2.92  7  8.07 | 13 (6/7)  13 (8/5)  14 (10/4) | 49.62  47.15  48.36 | US: hot pack, transcutaneous electrical nerve stimulation, and US therapy KT: hot pack , TENS  ESWT: hot pack, TENS | VAS | 2, 8 | KT+ |
| Rompe et al. 2003 | Yes | 15.9  12 | 40 (21/19)  35 (21/14) | 46.5  48.2 | ESWT: 1500 pulses at 0.18 mJ/mm2 Placebo: sham treatments | VAS | 12 | ESWT+ |
| Wong et al. 2005 | Yes | 11.83  19.07 | 30 (25/5)  30 (24/6) | 45.60  44.18 | BoNT-A: 60 units  Placebo: an equivalent volume of normal saline | VAS | 4, 12 | BoNT-A+ |
| Yalvaç et al. 2018 | Yes | 7.9  8.2 | 24 (16/8)  20 (15/5) | 43.75  46.04 | US: 1 cm2 application area, at 1.5 W/cm2, 1 MHz frequency, ESWT: 10-15 Hz, 1.5-2.5 bar energy, 2000 pulses | VAS | 4 | ESWT+ |
| Yerlikaya et al. 2018 | Yes | ＞3 | 30 (19/11)  30 (26/4)  30 (19/11) | 47.6  45.0  46.5 | Control: 1.5 ml saline LP-PRP1: 1.5 ml saline LR-PRP2: 1.5 ml saline | VAS | 4, 8 | No Difference |
| Hoseini et al. 2025 | Yes | 5.2  5.5  5.5 | 17 (12/5)  17 (11/6)  17 (9/8) | 43.58  45  42 | Brace: Y-shaped Kinesio strips Kinesio Taping: 1 ml of triamcinolone (20 mg/ml), 1 ml of 1% lidocaine Cortico steroid: Counterforce brace worn continuously for two weeks | VAS | 4 | All+ |
| Akkurt et al. 2025 | Yes | 7  12 | 21 (9/12)  21  (8/13) | 43.0  46.14 | KT Group: A standard 5-cm wide Kinesio® Tex Gold  Sham Group: non-allergic, non-elastic medical cloth tape | VAS | 3, 7 | KT+ |
| Kizilkurt et al. 2025 | Yes | > 3 | 12 (7/5)  12 (6/6)  12 (5/7) | 42.2 43.4  40.7 | Corticosteroid: 2 mL total (1 mL betamethasone + 1 mL bupivacaine HCl)  PRP: 2 mL PRP (from 16.2 mL blood + 1.8 mL sodium citrate) | VAS | 12, 24 | PRP+ |

“Diagnosis reported” indicates whether explicit diagnostic criteria for lateral epicondylitis were provided in the original study. Yes = reported; NR = not reported. F/M = female/male. Symptom duration is presented in months and follow-up in weeks. In the “Main result” column, “+” indicates that the intervention listed showed a more favorable pain-relieving effect than its comparator at the reported follow-up time point; “No difference” indicates that no statistically significant between-group difference was reported; “All” indicates improvement in all study arms without a clearly superior intervention.

VAS, visual analog scale; NR, not reported; HVLAT, high-velocity low-amplitude thrust; EX, exercise; DPT, dextrose prolotherapy; GAGPS, glycosaminoglycan polysulfate; US, ultrasound; CUS, continuous ultrasound; PUS, pulsed ultrasound; ESWT, extracorporeal shock wave therapy; WES, wrist extension splint; WAS, wait-and-see; PT, physical therapy; CI, corticosteroid injection; SI, steroid injection; CSI, corticosteroid injection; ASTM, augmented soft tissue mobilization; KT, kinesio taping; MET, muscle energy technique; LLLT, low-level laser therapy; HILT, high-intensity laser therapy; BoNT-A, botulinum toxin A; PRP, platelet-rich plasma; LP-PRP, leukocyte-poor platelet-rich plasma; LR-PRP, leukocyte-rich platelet-rich plasma.

Figure 2. Risk of bias assessment of the included studies. The upper panel presents the risk of bias judgments for each included study across the seven domains of the Cochrane risk of bias tool, while the lower panel summarizes the overall proportion of studies rated as low, unclear, or high risk of bias in each domain.







Figure 3. Network plots for short-term outcomes (1-4 weeks). Panel A shows the network structure weighted by the number of participants assigned to each intervention, with node size proportional to sample size. Panel B shows the network structure weighted by the number of direct comparisons, with edge thickness proportional to the number of studies contributing to each comparison.

B

A










Figure 4. Forest plot of short-term treatment effects on pain relief (1-4 weeks). The figure presents the relative effects of each intervention compared with placebo for short-term pain relief, expressed as mean differences (MDs) with 95% credible intervals (CrIs). Negative MD values indicate greater pain reduction and therefore a more favorable treatment effect.


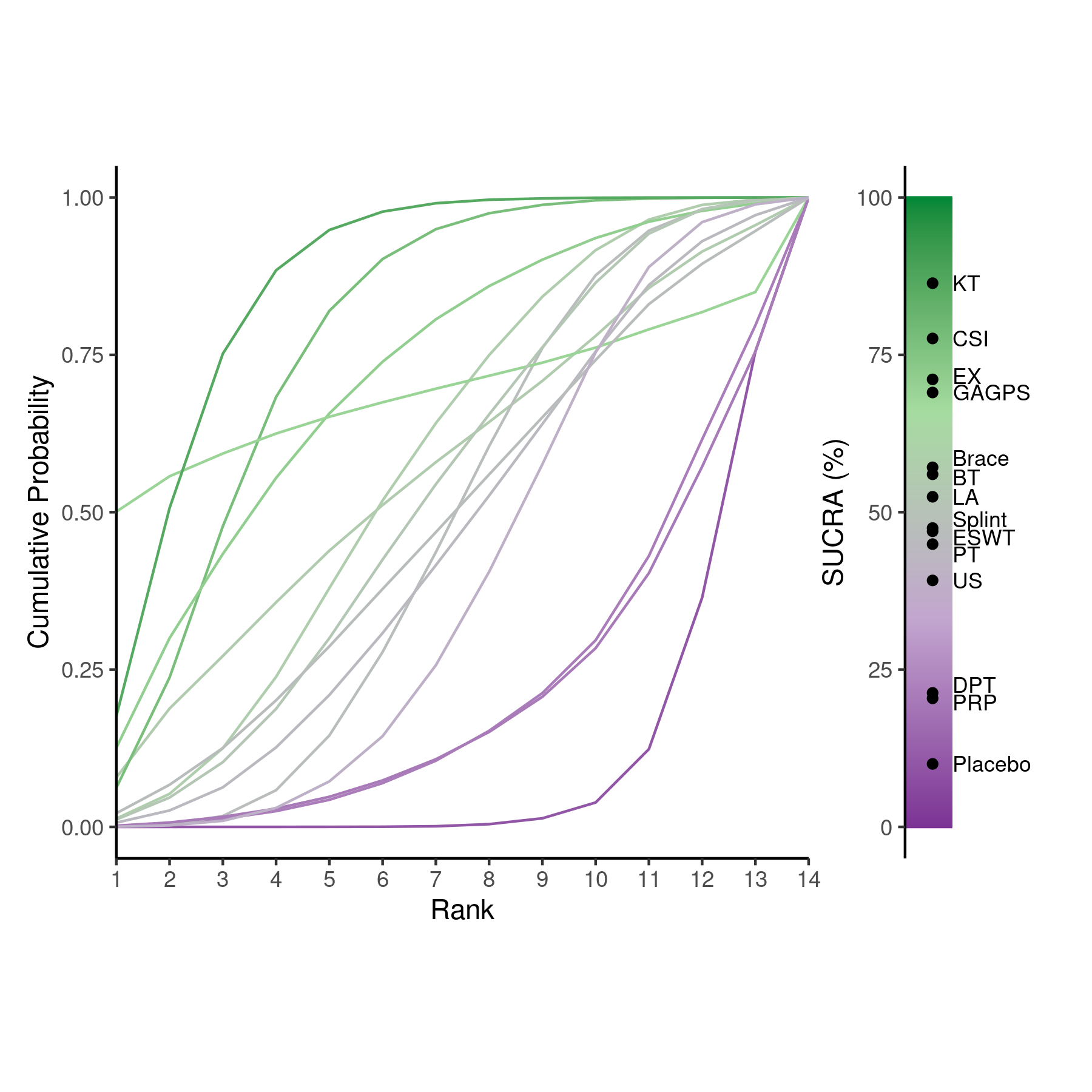


Figure 5. Cumulative ranking probability curves and SUCRA values for short-term pain relief (1-4 weeks). The cumulative ranking curves illustrate the probability of each intervention occupying each possible rank for short-term pain relief. SUCRA values summarize the overall ranking performance of each treatment, with higher values indicating a greater likelihood of better pain-relieving efficacy.





Figure 6. Node-splitting analysis for short-term outcomes (1-4 weeks). The figure presents the results of the node-splitting analysis used to assess local inconsistency between direct and indirect evidence in the short-term network. Comparisons with non-significant differences indicate no evidence of important inconsistency between direct and indirect estimates.

Table 2. League table of network meta-analysis results for short-term pain relief (1-4 weeks)

|  | **Brace** | **BT** | **CSI** | **DPT** | **ESWT** | **EX** | **GAGPS** | **KT** | **LA** | **Placebo** | **PRP** | **PT** | **Splint** | **US** |
| --- | --- | --- | --- | --- | --- | --- | --- | --- | --- | --- | --- | --- | --- | --- |
| **Brace** | **Brace** | -0.01 (-4.26, 4.23) | -1.07 (-3.57, 1.45) | 2.01 (-1.24, 5.31) | 0.47 (-2.3, 3.22) | -0.86 (-4.74, 2.97) | -2.34 (-13.09, 8.22) | -1.59 (-4.06, 0.86) | 0.24 (-2.07, 2.55) | 2.52 (0.42, 4.64) | 2.12 (-1.34, 5.56) | 0.59 (-2.57, 3.71) | 0.46 (-3.31, 4.21) | 0.82 (-1.81, 3.47) |
| **BT** | 0.01 (-4.23, 4.26) | **BT** | -1.05 (-5.33, 3.21) | 2.02 (-2.47, 6.54) | 0.49 (-3.88, 4.77) | -0.85 (-6.02, 4.23) | -2.33 (-13.56, 8.64) | -1.58 (-5.81, 2.63) | 0.24 (-3.98, 4.49) | 2.53 (-1.18, 6.22) | 2.11 (-2.45, 6.71) | 0.59 (-4, 5.14) | 0.49 (-4.58, 5.46) | 0.83 (-3.4, 5.05) |
| **CSI** | 1.07 (-1.45, 3.57) | 1.05 (-3.21, 5.33) | **CSI** | 3.07 (-0.22, 6.39) | 1.53 (-0.69, 3.73) | 0.2 (-3.32, 3.66) | -1.3 (-12.07, 9.35) | -0.53 (-2.33, 1.25) | 1.3 (-1.5, 4.1) | 3.57 (1.47, 5.71) | 3.18 (-0.28, 6.64) | 1.64 (-0.82, 4.1) | 1.52 (-1.87, 4.91) | 1.89 (-0.36, 4.14) |
| **DPT** | -2.01 (-5.31, 1.24) | -2.02 (-6.54, 2.47) | -3.07 (-6.39, 0.22) | **DPT** | -1.54 (-4.97, 1.8) | -2.88 (-7.2, 1.41) | -4.36 (-15.26, 6.28) | -3.6 (-6.85, -0.41) | -1.78 (-5.03, 1.44) | 0.5 (-2.02, 3.03) | 0.1 (-3.61, 3.76) | -1.43 (-5.15, 2.2) | -1.55 (-5.84, 2.66) | -1.19 (-4.43, 2.02) |
| **ESWT** | -0.47 (-3.22, 2.3) | -0.49 (-4.77, 3.88) | -1.53 (-3.73, 0.69) | 1.54 (-1.8, 4.97) | **ESWT** | -1.33 (-4.04, 1.36) | -2.81 (-13.67, 7.84) | -2.05 (-4.04, -0.05) | -0.23 (-3.17, 2.72) | 2.05 (-0.19, 4.32) | 1.66 (-1.9, 5.17) | 0.12 (-2.61, 2.83) | -0.01 (-2.57, 2.57) | 0.36 (-1.32, 2.06) |
| **EX** | 0.86 (-2.97, 4.74) | 0.85 (-4.23, 6.02) | -0.2 (-3.66, 3.32) | 2.88 (-1.41, 7.2) | 1.33 (-1.36, 4.04) | **EX** | -1.49 (-12.72, 9.45) | -0.73 (-4.07, 2.65) | 1.09 (-2.85, 5.11) | 3.38 (-0.09, 6.93) | 2.98 (-1.44, 7.44) | 1.44 (-2.35, 5.29) | 1.32 (-2.37, 5.04) | 1.68 (-1.48, 4.9) |
| **GAGPS** | 2.34 (-8.22, 13.09) | 2.33 (-8.64, 13.56) | 1.3 (-9.35, 12.07) | 4.36 (-6.28, 15.26) | 2.81 (-7.84, 13.67) | 1.49 (-9.45, 12.72) | **GAGPS** | 0.74 (-9.81, 11.58) | 2.57 (-7.96, 13.38) | 4.86 (-5.5, 15.48) | 4.46 (-6.22, 15.37) | 2.93 (-7.76, 13.88) | 2.79 (-8.12, 13.93) | 3.17 (-7.4, 13.97) |
| **KT** | 1.59 (-0.86, 4.06) | 1.58 (-2.63, 5.81) | 0.53 (-1.25, 2.33) | 3.6 (0.41, 6.85) | 2.05 (0.05, 4.04) | 0.73 (-2.65, 4.07) | -0.74 (-11.58, 9.81) | **KT** | 1.82 (-0.87, 4.56) | 4.1 (2.11, 6.14) | 3.7 (0.35, 7.1) | 2.17 (-0.55, 4.88) | 2.05 (-1.21, 5.32) | 2.41 (0.46, 4.39) |
| **LA** | -0.24 (-2.55, 2.07) | -0.24 (-4.49, 3.98) | -1.3 (-4.1, 1.5) | 1.78 (-1.44, 5.03) | 0.23 (-2.72, 3.17) | -1.09 (-5.11, 2.85) | -2.57 (-13.38, 7.96) | -1.82 (-4.56, 0.87) | **LA** | 2.28 (0.22, 4.36) | 1.88 (-1.53, 5.32) | 0.35 (-2.94, 3.61) | 0.22 (-3.69, 4.1) | 0.58 (-2.17, 3.39) |
| **Placebo** | -2.52 (-4.64, -0.42) | -2.53 (-6.22, 1.18) | -3.57 (-5.71, -1.47) | -0.5 (-3.03, 2.02) | -2.05 (-4.32, 0.19) | -3.38 (-6.93, 0.09) | -4.86 (-15.48, 5.5) | -4.1 (-6.14, -2.11) | -2.28 (-4.36, -0.22) | **Placebo** | -0.4 (-3.14, 2.32) | -1.93 (-4.63, 0.72) | -2.05 (-5.5, 1.35) | -1.69 (-3.71, 0.3) |
| **PRP** | -2.12 (-5.56, 1.34) | -2.11 (-6.71, 2.45) | -3.18 (-6.64, 0.28) | -0.1 (-3.76, 3.61) | -1.66 (-5.17, 1.9) | -2.98 (-7.44, 1.44) | -4.46 (-15.37, 6.22) | -3.7 (-7.1, -0.35) | -1.88 (-5.32, 1.53) | 0.4 (-2.32, 3.14) | **PRP** | -1.54 (-5.4, 2.27) | -1.66 (-6.01, 2.68) | -1.3 (-4.66, 2.09) |
| **PT** | -0.59 (-3.71, 2.57) | -0.59 (-5.14, 4) | -1.64 (-4.1, 0.82) | 1.43 (-2.2, 5.15) | -0.12 (-2.83, 2.61) | -1.44 (-5.29, 2.35) | -2.93 (-13.88, 7.76) | -2.17 (-4.88, 0.55) | -0.35 (-3.61, 2.94) | 1.93 (-0.72, 4.63) | 1.54 (-2.27, 5.4) | **PT** | -0.12 (-3.86, 3.63) | 0.24 (-2.55, 3.06) |
| **Splint** | -0.46 (-4.21, 3.31) | -0.49 (-5.46, 4.58) | -1.52 (-4.91, 1.87) | 1.55 (-2.66, 5.84) | 0.01 (-2.57, 2.57) | -1.32 (-5.04, 2.37) | -2.79 (-13.93, 8.12) | -2.05 (-5.32, 1.21) | -0.22 (-4.1, 3.69) | 2.05 (-1.35, 5.5) | 1.66 (-2.68, 6.01) | 0.12 (-3.63, 3.86) | **Splint** | 0.36 (-2.68, 3.43) |
| **US** | -0.82 (-3.47, 1.81) | -0.83 (-5.05, 3.4) | -1.89 (-4.14, 0.36) | 1.19 (-2.02, 4.43) | -0.36 (-2.06, 1.32) | -1.68 (-4.9, 1.48) | -3.17 (-13.97, 7.4) | -2.41 (-4.39, -0.46) | -0.58 (-3.39, 2.17) | 1.69 (-0.3, 3.71) | 1.3 (-2.09, 4.66) | -0.24 (-3.06, 2.55) | -0.36 (-3.43, 2.68) | **US** |

Results are expressed as mean differences (MDs) with 95% credible intervals (CrIs) for all pairwise comparisons. All pain scores were converted to a 0-10 VAS scale. Negative MD values favor the intervention listed in the row, indicating greater pain relief compared with the intervention listed in the column. Statistically significant comparisons are those with 95%CrIs excluding 0.

B

A

Figure 7. Network plots for intermediate-term outcomes (4-12 weeks). Panel A shows the network structure weighted by the number of direct comparisons, with edge thickness proportional to the number of studies contributing to each comparison. Panel B shows the network structure weighted by the number of participants assigned to each intervention, with node size proportional to sample size.










Figure 8. Forest plot of intermediate-term treatment effects on pain relief (4-12 weeks). The figure presents the relative effects of each intervention compared with placebo for intermediate-term pain relief, expressed as mean differences (MDs) with 95% credible intervals (CrIs). Negative MD values indicate greater pain reduction and therefore a more favorable treatment effect.


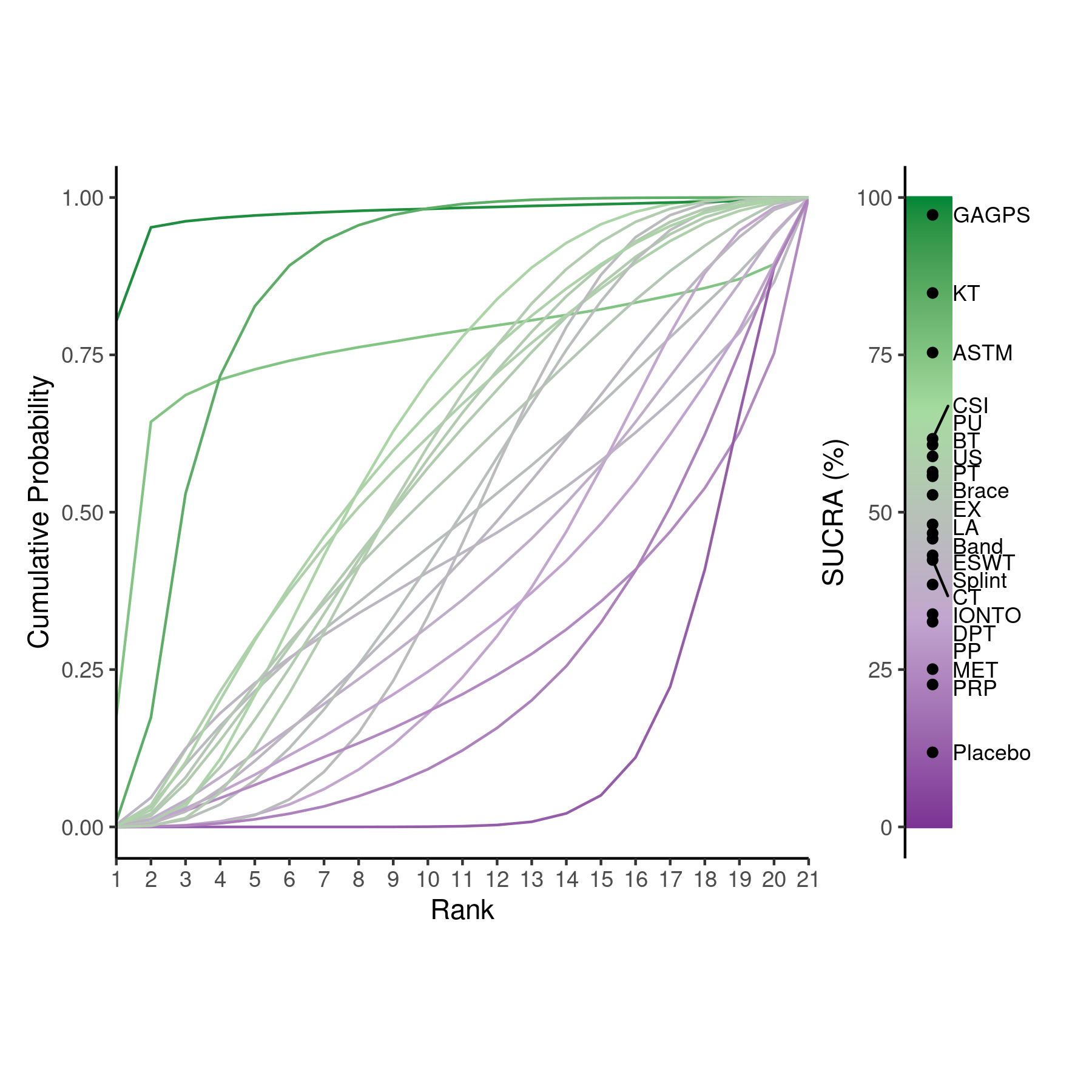


Figure 9. Cumulative ranking probability curves and SUCRA values for intermediate-term pain relief (4-12 weeks). The cumulative ranking curves illustrate the probability of each intervention occupying each possible rank for intermediate-term pain relief. SUCRA values summarize the overall ranking performance of each treatment, with higher values indicating a greater likelihood of better pain-relieving efficacy.





Figure 10. Node-splitting analysis for intermediate-term outcomes (4-12 weeks). The figure presents the results of the node-splitting analysis used to assess local inconsistency between direct and indirect evidence in the intermediate-term network. Comparisons with non-significant differences indicate no evidence of important inconsistency between direct and indirect estimates.

Table 3. League table of network meta-analysis results for intermediate-term pain relief (4-12 weeks)

|  | **ASTM** | **Band** | **Brace** | **BT** | **CSI** | **CT** | **DPT** | **ESWT** | **EX** | **GAGPS** | **IONTO** | **KT** | **LA** | **MET** | **Placebo** | **PP** | **PRP** | **PT** | **PU** | **Splint** | **US** |
| --- | --- | --- | --- | --- | --- | --- | --- | --- | --- | --- | --- | --- | --- | --- | --- | --- | --- | --- | --- | --- | --- |
| **ASTM** | **ASTM** | 3.43 (-5.53, 12.17) | 3.11 (-5.64, 11.73) | 2.98 (-5.78, 11.6) | 2.94 (-5.68, 11.48) | 3.62 (-5.61, 12.54) | 3.82 (-4.85, 12.28) | 3.41 (-5.24, 11.91) | 3.21 (-5.68, 11.87) | -5.35 (-17.9, 6.17) | 3.72 (-5.18, 12.43) | 1.97 (-6.67, 10.5) | 3.38 (-5.31, 11.9) | 4.36 (-4.59, 13.13) | 4.56 (-4.03, 13.01) | 3.94 (-4.93, 12.65) | 4.24 (-4.45, 12.81) | 3.12 (-5.58, 11.65) | 2.93 (-5.78, 11.49) | 3.53 (-5.29, 12.11) | 3.1 (-5.5, 11.61) |
| **Band** | -3.43 (-12.17, 5.53) | **Band** | -0.29 (-3.19, 2.56) | -0.44 (-3.48, 2.59) | -0.45 (-3.12, 2.17) | 0.19 (-3.58, 3.91) | 0.39 (-2.37, 3.12) | 0 (-2.27, 2.26) | -0.21 (-3.12, 2.65) | -8.91 (-17.86, -0.24) | 0.31 (-2.93, 3.46) | -1.43 (-4.19, 1.18) | -0.04 (-2.77, 2.65) | 0.94 (-2.6, 4.39) | 1.14 (-1.4, 3.68) | 0.53 (-2.76, 3.73) | 0.84 (-2.1, 3.67) | -0.3 (-3.01, 2.41) | -0.47 (-3.4, 2.41) | 0.11 (-1.53, 1.73) | -0.3 (-2.94, 2.31) |
| **Brace** | -3.11 (-11.73, 5.64) | 0.29 (-2.56, 3.19) | **Brace** | -0.14 (-2.45, 2.18) | -0.17 (-2.07, 1.76) | 0.47 (-2.97, 3.95) | 0.68 (-1.2, 2.61) | 0.29 (-1.46, 2.08) | 0.08 (-2.43, 2.6) | -8.61 (-17.37, -0.22) | 0.59 (-1.68, 2.86) | -1.15 (-3.15, 0.81) | 0.25 (-1.23, 1.74) | 1.23 (-1.78, 4.25) | 1.43 (-0.12, 3.02) | 0.81 (-1.49, 3.14) | 1.13 (-0.98, 3.18) | -0.01 (-2, 2.02) | -0.18 (-2.37, 2.01) | 0.39 (-1.96, 2.77) | -0.02 (-1.84, 1.84) |
| **BT** | -2.98 (-11.6, 5.78) | 0.44 (-2.59, 3.48) | 0.14 (-2.18, 2.45) | **BT** | -0.03 (-2.08, 2.03) | 0.62 (-2.97, 4.2) | 0.82 (-1.17, 2.82) | 0.44 (-1.57, 2.46) | 0.22 (-2.47, 2.89) | -8.47 (-17.23, -0.04) | 0.73 (-2.04, 3.48) | -1 (-3.16, 1.08) | 0.39 (-1.8, 2.55) | 1.37 (-1.73, 4.46) | 1.57 (-0.11, 3.28) | 0.96 (-1.85, 3.75) | 1.27 (-0.94, 3.43) | 0.13 (-2.01, 2.28) | -0.05 (-2.33, 2.26) | 0.54 (-2.04, 3.1) | 0.13 (-1.85, 2.12) |
| **CSI** | -2.94 (-11.48, 5.68) | 0.45 (-2.17, 3.12) | 0.17 (-1.76, 2.07) | 0.03 (-2.03, 2.08) | **CSI** | 0.64 (-2.61, 3.92) | 0.84 (-0.73, 2.44) | 0.47 (-0.88, 1.81) | 0.24 (-1.97, 2.47) | -8.44 (-17.12, -0.11) | 0.76 (-1.68, 3.17) | -0.98 (-2.32, 0.29) | 0.42 (-1.3, 2.12) | 1.4 (-0.94, 3.71) | 1.6 (0.44, 2.77) | 0.98 (-1.5, 3.44) | 1.29 (-0.48, 3.04) | 0.16 (-1.12, 1.45) | -0.02 (-1.88, 1.84) | 0.56 (-1.51, 2.63) | 0.15 (-1.23, 1.57) |
| **CT** | -3.62 (-12.54, 5.61) | -0.19 (-3.91, 3.58) | -0.47 (-3.95, 2.97) | -0.62 (-4.2, 2.97) | -0.64 (-3.92, 2.61) | **CT** | 0.21 (-3.13, 3.55) | -0.18 (-3.15, 2.79) | -0.4 (-2.78, 1.98) | -9.09 (-18.27, -0.2) | 0.12 (-3.63, 3.82) | -1.62 (-4.99, 1.63) | -0.22 (-3.59, 3.07) | 0.75 (-3.24, 4.77) | 0.96 (-2.22, 4.13) | 0.34 (-3.41, 4.09) | 0.65 (-2.83, 4.07) | -0.48 (-3.82, 2.82) | -0.67 (-4.14, 2.8) | -0.08 (-3.42, 3.29) | -0.49 (-3.74, 2.74) |
| **DPT** | -3.82 (-12.28, 4.85) | -0.39 (-3.12, 2.37) | -0.68 (-2.61, 1.2) | -0.82 (-2.82, 1.17) | -0.84 (-2.44, 0.73) | -0.21 (-3.55, 3.13) | **DPT** | -0.38 (-1.91, 1.14) | -0.61 (-2.94, 1.73) | -9.28 (-17.98, -1.01) | -0.09 (-2.54, 2.34) | -1.83 (-3.54, -0.21) | -0.42 (-2.18, 1.29) | 0.54 (-2.27, 3.34) | 0.75 (-0.31, 1.82) | 0.14 (-2.36, 2.61) | 0.45 (-1.32, 2.16) | -0.69 (-2.4, 1.01) | -0.87 (-2.76, 0.99) | -0.29 (-2.48, 1.89) | -0.7 (-2.18, 0.8) |
| **ESWT** | -3.41 (-11.91, 5.24) | 0 (-2.26, 2.27) | -0.29 (-2.08, 1.46) | -0.44 (-2.46, 1.57) | -0.47 (-1.81, 0.88) | 0.18 (-2.79, 3.15) | 0.38 (-1.14, 1.91) | **ESWT** | -0.22 (-2.01, 1.56) | -8.91 (-17.56, -0.55) | 0.3 (-1.98, 2.53) | -1.44 (-2.9, -0.07) | -0.04 (-1.53, 1.41) | 0.93 (-1.77, 3.59) | 1.14 (0.04, 2.24) | 0.53 (-1.8, 2.82) | 0.83 (-0.96, 2.57) | -0.31 (-1.78, 1.19) | -0.49 (-2.28, 1.32) | 0.1 (-1.47, 1.67) | -0.31 (-1.59, 0.98) |
| **EX** | -3.21 (-11.87, 5.68) | 0.21 (-2.65, 3.12) | -0.08 (-2.6, 2.43) | -0.22 (-2.89, 2.47) | -0.24 (-2.47, 1.97) | 0.4 (-1.98, 2.78) | 0.61 (-1.73, 2.94) | 0.22 (-1.56, 2.01) | **EX** | -8.68 (-17.55, -0.15) | 0.52 (-2.37, 3.38) | -1.22 (-3.53, 1.02) | 0.18 (-2.15, 2.48) | 1.15 (-2.07, 4.37) | 1.36 (-0.72, 3.45) | 0.75 (-2.19, 3.67) | 1.05 (-1.46, 3.52) | -0.08 (-2.4, 2.21) | -0.26 (-2.79, 2.25) | 0.32 (-2.03, 2.69) | -0.09 (-2.28, 2.11) |
| **GAGPS** | 5.35 (-6.17, 17.9) | 8.91 (0.24, 17.86) | 8.61 (0.22, 17.37) | 8.47 (0.04, 17.23) | 8.44 (0.11, 17.12) | 9.09 (0.2, 18.27) | 9.28 (1.01, 17.98) | 8.91 (0.55, 17.56) | 8.68 (0.15, 17.55) | **GAGPS** | 9.21 (0.66, 18.04) | 7.45 (-0.84, 16.14) | 8.86 (0.49, 17.53) | 9.84 (1.16, 18.82) | 10.04 (1.81, 18.64) | 9.42 (0.86, 18.3) | 9.73 (1.42, 18.46) | 8.61 (0.22, 17.31) | 8.43 (0.03, 17.17) | 9.01 (0.5, 17.77) | 8.6 (0.3, 17.28) |
| **IONTO** | -3.72 (-12.43, 5.18) | -0.31 (-3.46, 2.93) | -0.59 (-2.86, 1.68) | -0.73 (-3.48, 2.04) | -0.76 (-3.17, 1.68) | -0.12 (-3.82, 3.63) | 0.09 (-2.34, 2.54) | -0.3 (-2.53, 1.98) | -0.52 (-3.38, 2.37) | -9.21 (-18.04, -0.66) | **IONTO** | -1.74 (-4.24, 0.7) | -0.34 (-2.06, 1.35) | 0.63 (-2.72, 4) | 0.84 (-1.33, 3.04) | 0.22 (-1.5, 1.95) | 0.54 (-2.08, 3.09) | -0.6 (-3.09, 1.92) | -0.78 (-3.43, 1.89) | -0.2 (-2.93, 2.55) | -0.61 (-2.95, 1.77) |
| **KT** | -1.97 (-10.5, 6.67) | 1.43 (-1.18, 4.19) | 1.15 (-0.81, 3.15) | 1 (-1.08, 3.16) | 0.98 (-0.29, 2.32) | 1.62 (-1.63, 4.99) | 1.83 (0.21, 3.54) | 1.44 (0.07, 2.9) | 1.22 (-1.02, 3.53) | -7.45 (-16.14, 0.84) | 1.74 (-0.7, 4.24) | **KT** | 1.39 (-0.35, 3.22) | 2.37 (-0.25, 5.06) | 2.58 (1.32, 3.92) | 1.97 (-0.54, 4.52) | 2.27 (0.43, 4.15) | 1.14 (-0.45, 2.81) | 0.96 (-0.87, 2.88) | 1.53 (-0.53, 3.71) | 1.13 (-0.12, 2.5) |
| **LA** | -3.38 (-11.9, 5.31) | 0.04 (-2.65, 2.77) | -0.25 (-1.74, 1.23) | -0.39 (-2.55, 1.8) | -0.42 (-2.12, 1.3) | 0.22 (-3.07, 3.59) | 0.42 (-1.29, 2.18) | 0.04 (-1.41, 1.53) | -0.18 (-2.48, 2.15) | -8.86 (-17.53, -0.49) | 0.34 (-1.35, 2.06) | -1.39 (-3.22, 0.35) | **LA** | 0.98 (-1.9, 3.86) | 1.18 (-0.17, 2.56) | 0.57 (-1.2, 2.34) | 0.88 (-1.08, 2.8) | -0.26 (-2.07, 1.58) | -0.44 (-2.45, 1.61) | 0.14 (-2, 2.31) | -0.26 (-1.89, 1.39) |
| **MET** | -4.36 (-13.13, 4.59) | -0.94 (-4.39, 2.6) | -1.23 (-4.25, 1.78) | -1.37 (-4.46, 1.73) | -1.4 (-3.71, 0.94) | -0.75 (-4.77, 3.24) | -0.54 (-3.34, 2.27) | -0.93 (-3.59, 1.77) | -1.15 (-4.37, 2.07) | -9.84 (-18.82, -1.16) | -0.63 (-4, 2.72) | -2.37 (-5.06, 0.25) | -0.98 (-3.86, 1.9) | **MET** | 0.2 (-2.38, 2.81) | -0.42 (-3.81, 2.97) | -0.11 (-3.03, 2.78) | -1.24 (-3.86, 1.43) | -1.42 (-4.36, 1.58) | -0.83 (-3.93, 2.3) | -1.24 (-3.95, 1.48) |
| **Placebo** | -4.56 (-13.01, 4.03) | -1.14 (-3.68, 1.4) | -1.43 (-3.02, 0.12) | -1.57 (-3.28, 0.11) | -1.6 (-2.77, -0.44) | -0.96 (-4.13, 2.22) | -0.75 (-1.82, 0.31) | -1.14 (-2.24, -0.04) | -1.36 (-3.45, 0.72) | -10.04 (-18.64, -1.81) | -0.84 (-3.04, 1.33) | -2.58 (-3.92, -1.32) | -1.18 (-2.56, 0.17) | -0.2 (-2.81, 2.38) | **Placebo** | -0.62 (-2.87, 1.62) | -0.3 (-1.71, 1.06) | -1.44 (-2.77, -0.12) | -1.62 (-3.17, -0.07) | -1.04 (-2.97, 0.88) | -1.44 (-2.48, -0.4) |
| **PP** | -3.94 (-12.65, 4.93) | -0.53 (-3.73, 2.76) | -0.81 (-3.14, 1.49) | -0.96 (-3.75, 1.85) | -0.98 (-3.44, 1.5) | -0.34 (-4.09, 3.41) | -0.14 (-2.61, 2.36) | -0.53 (-2.82, 1.8) | -0.75 (-3.67, 2.19) | -9.42 (-18.3, -0.86) | -0.22 (-1.95, 1.5) | -1.97 (-4.52, 0.54) | -0.57 (-2.34, 1.2) | 0.42 (-2.97, 3.81) | 0.62 (-1.62, 2.87) | **PP** | 0.31 (-2.37, 2.93) | -0.83 (-3.36, 1.74) | -1 (-3.71, 1.7) | -0.42 (-3.19, 2.38) | -0.83 (-3.23, 1.6) |
| **PRP** | -4.24 (-12.81, 4.45) | -0.84 (-3.67, 2.1) | -1.13 (-3.18, 0.98) | -1.27 (-3.43, 0.94) | -1.29 (-3.04, 0.48) | -0.65 (-4.07, 2.83) | -0.45 (-2.16, 1.32) | -0.83 (-2.57, 0.96) | -1.05 (-3.52, 1.46) | -9.73 (-18.46, -1.42) | -0.54 (-3.09, 2.08) | -2.27 (-4.15, -0.43) | -0.88 (-2.8, 1.08) | 0.11 (-2.78, 3.03) | 0.3 (-1.06, 1.71) | -0.31 (-2.93, 2.37) | **PRP** | -1.13 (-2.99, 0.76) | -1.31 (-3.35, 0.76) | -0.73 (-3.07, 1.67) | -1.14 (-2.83, 0.61) |
| **PT** | -3.12 (-11.65, 5.58) | 0.3 (-2.41, 3.01) | 0.01 (-2.02, 2) | -0.13 (-2.28, 2.01) | -0.16 (-1.45, 1.12) | 0.48 (-2.82, 3.82) | 0.69 (-1.01, 2.4) | 0.31 (-1.19, 1.78) | 0.08 (-2.21, 2.4) | -8.61 (-17.31, -0.22) | 0.6 (-1.92, 3.09) | -1.14 (-2.81, 0.45) | 0.26 (-1.58, 2.07) | 1.24 (-1.43, 3.86) | 1.44 (0.12, 2.77) | 0.83 (-1.74, 3.36) | 1.13 (-0.76, 2.99) | **PT** | -0.18 (-2.15, 1.8) | 0.41 (-1.76, 2.56) | 0 (-1.58, 1.59) |
| **PU** | -2.93 (-11.49, 5.78) | 0.47 (-2.41, 3.4) | 0.18 (-2.01, 2.37) | 0.05 (-2.26, 2.33) | 0.02 (-1.84, 1.88) | 0.67 (-2.8, 4.14) | 0.87 (-0.99, 2.76) | 0.49 (-1.32, 2.28) | 0.26 (-2.25, 2.79) | -8.43 (-17.17, -0.03) | 0.78 (-1.89, 3.43) | -0.96 (-2.88, 0.87) | 0.44 (-1.61, 2.45) | 1.42 (-1.58, 4.36) | 1.62 (0.07, 3.17) | 1 (-1.7, 3.71) | 1.31 (-0.76, 3.35) | 0.18 (-1.8, 2.15) | **PU** | 0.58 (-1.8, 2.97) | 0.17 (-1.34, 1.71) |
| **Splint** | -3.53 (-12.11, 5.29) | -0.11 (-1.73, 1.53) | -0.39 (-2.77, 1.96) | -0.54 (-3.1, 2.04) | -0.56 (-2.63, 1.51) | 0.08 (-3.29, 3.42) | 0.29 (-1.89, 2.48) | -0.1 (-1.67, 1.47) | -0.32 (-2.69, 2.03) | -9.01 (-17.77, -0.5) | 0.2 (-2.55, 2.93) | -1.53 (-3.71, 0.53) | -0.14 (-2.31, 2) | 0.83 (-2.3, 3.93) | 1.04 (-0.88, 2.97) | 0.42 (-2.38, 3.19) | 0.73 (-1.67, 3.07) | -0.41 (-2.56, 1.76) | -0.58 (-2.97, 1.8) | **Splint** | -0.41 (-2.42, 1.62) |
| **US** | -3.1 (-11.61, 5.5) | 0.3 (-2.31, 2.94) | 0.02 (-1.84, 1.84) | -0.13 (-2.12, 1.85) | -0.15 (-1.57, 1.23) | 0.49 (-2.74, 3.74) | 0.7 (-0.8, 2.18) | 0.31 (-0.98, 1.59) | 0.09 (-2.11, 2.28) | -8.6 (-17.28, -0.3) | 0.61 (-1.77, 2.95) | -1.13 (-2.5, 0.12) | 0.26 (-1.39, 1.89) | 1.24 (-1.48, 3.95) | 1.44 (0.4, 2.48) | 0.83 (-1.6, 3.23) | 1.14 (-0.61, 2.83) | 0 (-1.59, 1.58) | -0.17 (-1.71, 1.34) | 0.41 (-1.62, 2.42) | **US** |

Results are presented as mean differences (MDs) with 95% credible intervals (CrIs) for pairwise comparisons between interventions. All pain outcomes were standardized to a 0-10 visual analog scale (VAS). A negative MD indicates that the treatment listed in the row was associated with greater pain reduction than the treatment listed in the column. Comparisons with 95%CrIs not crossing 0 were considered statistically significant.

B

A

Figure 11. Network plots for long-term outcomes (＞12 weeks). Panel A shows the network structure weighted by the number of direct comparisons, with edge thickness proportional to the number of studies contributing to each comparison. Panel B shows the network structure weighted by the number of participants assigned to each intervention, with node size proportional to sample size.










Figure 12. Forest plot of long-term treatment effects on pain relief (＞12 weeks). The figure presents the relative effects of each intervention compared with placebo for long-term pain relief, expressed as mean differences (MDs) with 95% credible intervals (CrIs). Negative MD values indicate greater pain reduction and therefore a more favorable treatment effect.


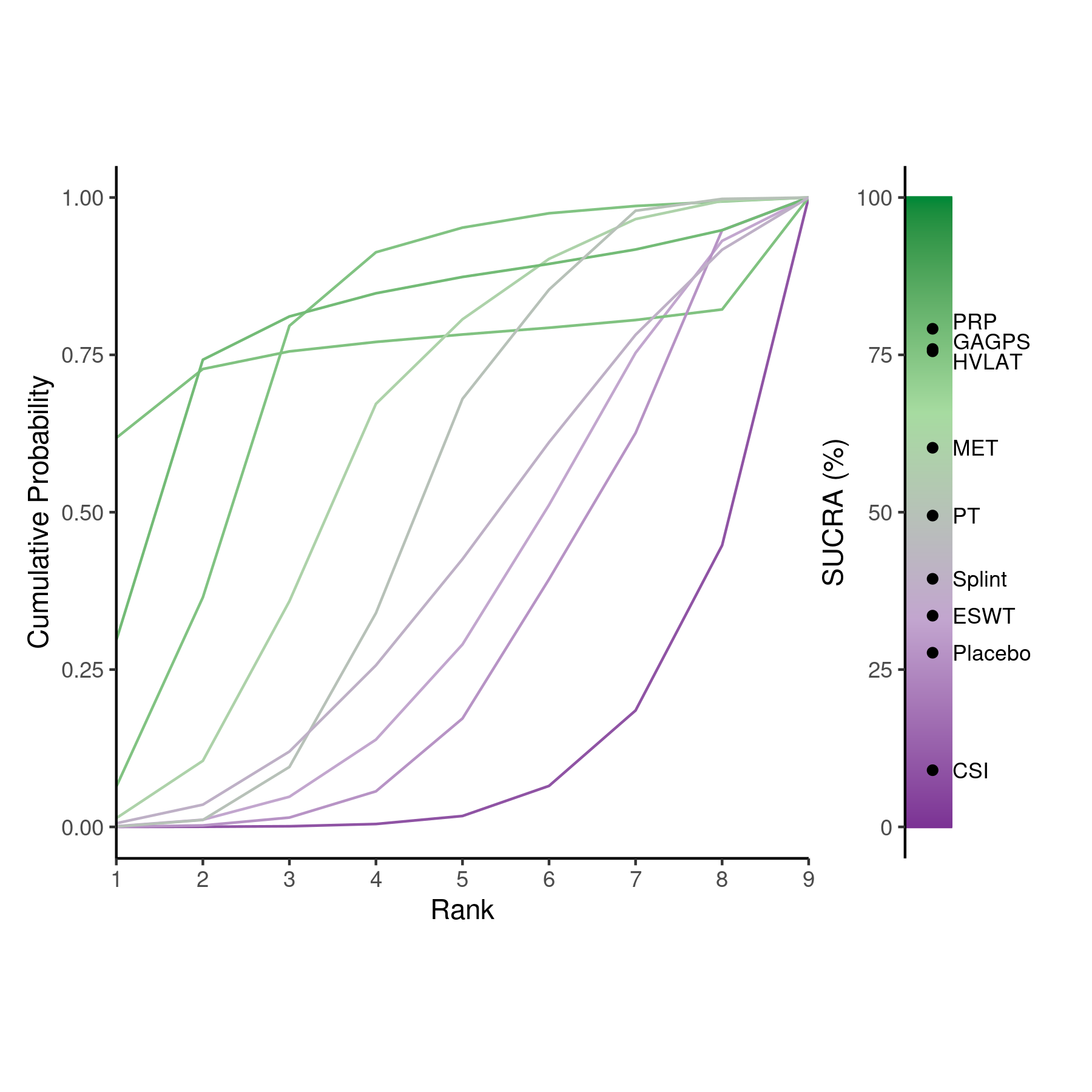


Figure 13. Cumulative ranking probability curves and SUCRA values for long-term pain relief (＞12 weeks). The cumulative ranking curves illustrate the probability of each intervention occupying each possible rank for long-term pain relief. SUCRA values summarize the overall ranking performance of each treatment, with higher values indicating a greater likelihood of better pain-relieving efficacy.





Figure 14. Node-splitting analysis for long-term outcomes (＞12 weeks). The figure presents the results of the node-splitting analysis used to assess local inconsistency between direct and indirect evidence in the long-term network. Comparisons with non-significant differences indicate no evidence of important inconsistency between direct and indirect estimates.

Table 4. League table of network meta-analysis results for long-term pain relief (＞12 weeks)

|  | **CSI** | **ESWT** | **GAGPS** | **HVLAT** | **MET** | **Placebo** | **PRP** | **PT** | **Splint** |
| --- | --- | --- | --- | --- | --- | --- | --- | --- | --- |
| **CSI** | **CSI** | -0.73 (-2.16, 0.93) | -6.61 (-19.69, 8.21) | -2.12 (-3.71, -0.37) | -1.45 (-2.63, -0.32) | -0.57 (-1.5, 0.48) | -3.73 (-8.92, 1.1) | -1.11 (-1.89, -0.16) | -0.84 (-2.56, 1.11) |
| **ESWT** | 0.73 (-0.93, 2.16) | **ESWT** | -5.92 (-19.04, 8.94) | -1.38 (-3.51, 0.59) | -0.73 (-2.78, 1.05) | 0.16 (-1.62, 1.78) | -3.04 (-8.44, 2) | -0.38 (-1.92, 1.08) | -0.11 (-1.12, 0.88) |
| **GAGPS** | 6.61 (-8.21, 19.69) | 5.92 (-8.94, 19.04) | **GAGPS** | 4.54 (-10.33, 17.65) | 5.16 (-9.67, 18.17) | 6.06 (-8.76, 19.12) | 2.79 (-12.61, 17.14) | 5.53 (-9.26, 18.59) | 5.81 (-9.13, 18.95) |
| **HVLAT** | 2.12 (0.37, 3.71) | 1.38 (-0.59, 3.51) | -4.54 (-17.65, 10.33) | **HVLAT** | 0.66 (-1.45, 2.6) | 1.55 (-0.19, 3.23) | -1.61 (-6.99, 3.41) | 1 (-0.39, 2.44) | 1.27 (-0.96, 3.61) |
| **MET** | 1.45 (0.32, 2.63) | 0.73 (-1.05, 2.78) | -5.16 (-18.17, 9.67) | -0.66 (-2.6, 1.45) | **MET** | 0.88 (-0.56, 2.47) | -2.29 (-7.62, 2.7) | 0.34 (-1.02, 1.88) | 0.62 (-1.42, 2.91) |
| **Placebo** | 0.57 (-0.48, 1.5) | -0.16 (-1.78, 1.62) | -6.06 (-19.12, 8.76) | -1.55 (-3.23, 0.19) | -0.88 (-2.47, 0.56) | **Placebo** | -3.18 (-8.35, 1.65) | -0.54 (-1.47, 0.44) | -0.28 (-2.17, 1.77) |
| **PRP** | 3.73 (-1.1, 8.92) | 3.04 (-2, 8.44) | -2.79 (-17.14, 12.61) | 1.61 (-3.41, 6.99) | 2.29 (-2.7, 7.62) | 3.18 (-1.65, 8.35) | **PRP** | 2.65 (-2.2, 7.86) | 2.91 (-2.18, 8.42) |
| **PT** | 1.11 (0.16, 1.89) | 0.38 (-1.08, 1.92) | -5.53 (-18.59, 9.26) | -1 (-2.44, 0.39) | -0.34 (-1.88, 1.02) | 0.54 (-0.44, 1.47) | -2.65 (-7.86, 2.2) | **PT** | 0.27 (-1.51, 2.11) |
| **Splint** | 0.84 (-1.11, 2.56) | 0.11 (-0.88, 1.12) | -5.81 (-18.95, 9.13) | -1.27 (-3.61, 0.96) | -0.62 (-2.91, 1.42) | 0.28 (-1.77, 2.17) | -2.91 (-8.42, 2.18) | -0.27 (-2.11, 1.51) | **Splint** |

Results are presented as mean differences (MDs)with 95% credible intervals (CrIs) for pairwise comparisons between interventions. All pain outcomes were standardized to a 0-10 visual analog scale (VAS). A negative MD indicates that the treatment listed in the row was associated with greater pain reduction than the treatment listed in the column. Comparisons with 95%CrIs not crossing 0 were considered statistically significant.

Supplementary Table S1. Baseline characteristics relevant to the transitivity assessment

| **Study key** | **Study full** | **T** | **N** | **Baseline (mean)** | **Baseline (sd)** | **Years (mean)** | **Years (ad)** | **Male** | **Female** | **Duration (month)** | **VAS (scale)** | **Co** |
| --- | --- | --- | --- | --- | --- | --- | --- | --- | --- | --- | --- | --- |
| Agarwal | Agarwal et al. 2023 | HVLAT | 10 | 8 | 0.66 | 30-45 | NR | NR | NR | NR | 10 | NO |
| Agarwal | Agarwal et al. 2023 | PT | 10 | 8.1 | 0.73 | 30-45 | NR | NR | NR | NR | 10 | NO |
| Agostinucci | Agostinucci et al. 2012 | CT | 19 | NR | NR | NR | NR | NR | NR | 3 | 10 | NO |
| Agostinucci | Agostinucci et al. 2012 | EX | 9 | NR | NR | NR | NR | NR | NR | 3 | 10 | NO |
| Akcay | Akcay et al. 2020 | DPT | 23 | 6 | 0.75 | 48.1 | 8.9 | 5 (22%) | 18 (78%) | 3 | 10 | NO |
| Akcay | Akcay et al. 2020 | Placebo | 27 | 5.5 | 0.5 | 46.7 | 8.3 | 8 (30%) | 19 (70%) | 3 | 10 | NO |
| Akermark | Akermark et al.,1995 | GAGPS | 15 | 61.7 | 16.2 | 46 | 6.75 | 15 (50%) | 15 (50%) | 3-36 | 100 | NO |
| Akermark | Akermark et al.,1995 | Placebo | 15 | 55.8 | 21 | 42 | 7.75 | 16 (53%) | 14 (47%) | 3-30 | 100 | NO |
| Akin | Akin et al. 2010 | Placebo | 30 | 2.4 | 2.1 | 45.4 | 8.1 | 10 (34%) | 20 (66%) | 6.8 | 10 | NO |
| Akin | Akin et al. 2010 | US | 30 | 3.5 | 2.7 | 46.7 | 8.1 | 12 (40%) | 18 (60%) | 8.7 | 10 | NO |
| Aydin | Aydin et al. 2018 | ESWT | 32 | 4.7 | 1.5 | 38.84 | 6.77 | 15 (47%) | 17 (53%) | 1 | 10 | NO |
| Aydin | Aydin et al. 2018 | Splint | 35 | 4.7 | 1.5 | 37.94 | 6.45 | 19 (54%) | 16 (46%) | 1 | 10 | NO |
| Baktir | Baktir et al. 2019 | IONTO | 13 | 5.31 | 3.14 | 49.31 | 9.23 | 3 (2%) | 10 (98%) | 12 | 10 | NO |
| Baktir | Baktir et al. 2019 | LA | 12 | 6.08 | 2.06 | 45.33 | 6.22 | 2 (17%) | 10 (83%) | 11 | 10 | NO |
| Baktir | Baktir et al. 2019 | PP | 12 | 3.17 | 2.25 | 43.75 | 7.94 | 3 (25%) | 9 (75%) | 12.25 | 10 | NO |
| Bisset | Bisset et al. 2006 | CSI | 65 | 53.5 | 23 | 47.8 | 8.2 | 40 (61%) | 25 (39%) | 3.7 | 100 | NO |
| Bisset | Bisset et al. 2006 | PT | 66 | 57.5 | 25 | 47.9 | 7.2 | 45 (68%) | 21 (32%) | 6.06 | 100 | NO |
| Bisset | Bisset et al. 2006 | Placebo | 67 | 61.3 | 22.6 | 47.3 | 8.1 | 43 (64%) | 24 (36%) | 6.06 | 100 | NO |
| Blanchette | Blanchette et al. 2011 | ASTM | 12 | 46 | 23 | 47 | 10 | 6 (40%) | 9 (60%) | 22 | 100 | NO |
| Blanchette | Blanchette et al. 2011 | Placebo | 15 | 39 | 29 | 46 | 10 | 6 (50%) | 6 (50%) | 43 | 100 | NO |
| Creuze | Creuze et al. 2018 | BT | 29 | 56.4 | 19 | 47.3 | 7 | 17 (57%) | 13 (43%) | 17.2 | 100 | NO |
| Creuze | Creuze et al. 2018 | Placebo | 28 | 55.8 | 20 | 46.7 | 6 | 16 (53%) | 14 (47%) | 20.2 | 100 | NO |
| Devrimsel | Devrimsel et al. 2014 | ESWT | 30 | 6.65 | 1.22 | 37.76 | 8.52 | 8 (27%) | 22 (73%) | NR | 10 | NO |
| Devrimsel | Devrimsel et al. 2014 | LA | 30 | 6.56 | 1.3 | 40.3 | 10 | 10 (33%) | 20 (67%) | NR | 10 | NO |
| Dundar | Dundar et al. 2015 | HILT | 30 | 4.3 | 1.3 | 32.6 | 10.9 | 13 (43%) | 17 (57%) | 28.7 | 10 | NO |
| Dundar | Dundar et al. 2015 | Brace | 30 | 4.2 | 1.5 | 33.6 | 9.8 | 15 (50%) | 15 (50%) | 27.9 | 10 | NO |
| Dundar | Dundar et al. 2015 | Placebo | 31 | 4.4 | 1.2 | 33.4 | 11.2 | 14 (45%) | 17 (55%) | 29.5 | 10 | NO |
| Gndz | Gündüz et al. 2012 | CSI | 20 | 8 | 1 | 45.7 | 10.2 | 8 (40%) | 12 (60%) | 3 | 10 | NO |
| Gndz | Gündüz et al. 2012 | ESWT | 20 | 8 | 0.75 | 44.9 | 9.9 | 5 (26%) | 14 (74%) | 3 | 10 | NO |
| Gndz | Gündüz et al. 2012 | PT | 19 | 8 | 1 | 43.6 | 9.1 | 5 (26%) | 14 (74%) | 3 | 10 | NO |
| Huseyin | Huseyin et al. 2021 | PU | 17 | 3 | 1.2 | 47.2 | 4.2 | 5 (29%) | 12 (71%) | 3.7 | 10 | NO |
| Huseyin | Huseyin et al. 2021 | Placebo | 17 | 7.8 | 1.8 | 47.1 | 6.8 | 6 (35%) | 11 (65%) | 3.7 | 10 | NO |
| Huseyin | Huseyin et al. 2021 | US | 17 | 2.9 | 0.8 | 45.3 | 7.5 | 7 (41%) | 10 (59%) | 3.5 | 10 | NO |
| Kksen | Küçüksen et al. 2013 | CSI | 41 | 7.17 | 1.07 | 43.78 | 9.16 | 19 (46%) | 22 (54%) | 6.1 | 10 | NO |
| Kksen | Küçüksen et al. 2013 | MET | 41 | 7.39 | 1.07 | 46.17 | 7.56 | 18 (43%) | 23 (57%) | 5.3 | 10 | NO |
| Koak | Koçak et al. 2019 | CSI | 28 | 2.79 | 2.45 | 43.54 | 12.08 | 14 (50%) | 14 (50%) | 4.50 | 10 | NO |
| Koak | Koçak et al. 2019 | KT | 28 | 1.93 | 1.56 | 40.96 | 11.6 | 10 (36%) | 18 (64%) | 5.07 | 10 | NO |
| Lam | Lam et al. 2007 | LA | 21 | 1.52 | 0.89 | 46.1 | 9.2 | 9 (43%) | 12 (57%) | 3.2 | 10 | NO |
| Lam | Lam et al. 2007 | Placebo | 18 | 5.14 | 1.88 | 48.9 | 8.7 | 7 (39%) | 11 (61%) | 3.3 | 10 | NO |
| Montalvan | Montalvan et al. 2016 | PRP | 25 | 6.8 | 0.8 | 35-65 | NR | NR | NR | 3 | 10 | NO |
| Montalvan | Montalvan et al. 2016 | Placebo | 25 | 7 | 1 | 35-65 | NR | NR | NR | 3 | 10 | NO |
| Rompe | Rompe et al. 2003 | ESWT | 40 | NR | NR | 46.5 | NR | 19 (48%) | 21 (52%) | 15.9 | 10 | NO |
| Rompe | Rompe et al. 2003 | Placebo | 35 | NR | NR | 48.2 | NR | 14 (40%) | 21 (60%) | 12 | 10 | NO |
| Wong | Wong et al. 2005 | BT | 30 | 65.5 | 15 | 45.6 | 9.06 | 5 (17%) | 25 (83%) | 11.83 | 100 | NO |
| Wong | Wong et al. 2005 | Placebo | 30 | 66.2 | 13.2 | 44.18 | 5.72 | 6 (20%) | 24 (80%) | 19.07 | 100 | NO |
| Yalva | Yalvaç et al. 2018 | ESWT | 20 | 3 | 1.5 | 46.04 | 9.24 | 8 (34%) | 16 (66%) | 8.2 | 10 | NO |
| Yalva | Yalvaç et al. 2018 | US | 24 | 7 | 1.75 | 43.75 | 4.52 | 5 (25%) | 15 (75%) | 7.9 | 10 | NO |
| Yerlikaya | Yerlikaya et al. 2018 | PRP | 30 | 7.4 | 2.1 | 45 | 8.6 | 4 (13%) | 26 (77%) | 3 | 10 | NO |
| Yerlikaya | Yerlikaya et al. 2018 | Placebo | 30 | 6.8 | 1.8 | 47.6 | 9.1 | 11 (37%) | 19 (63%) | 3 | 10 | NO |
| orum | Çorum et al. 2021 | ESWT | 22 | 5 | 2.25 | 49 | 23-57 | 6 (27%) | 16 (73%) | 3 | 10 | NO |
| orum | Çorum et al. 2021 | EX | 19 | 4 | 1.25 | 45 | 28-55 | 5 (26%) | 14 (74%) | 3 | 10 | NO |
| zmen | Özmen et al. 2021 | ESWT | 14 | 7 | 3 | 48.36 | 11.51 | 4 (29%) | 10 (71%) | 2.92 | 10 | NO |
| zmen | Özmen et al. 2021 | KT | 13 | 3.21 | 3.04 | 47.15 | 9.87 | 5 (39%) | 8 (62%) | 7 | 10 | NO |
| zmen | Özmen et al. 2021 | US | 13 | 1.46 | 0.32 | 49.62 | 10.2 | 7 (54%) | 6 (46%) | 8.07 | 10 | NO |
| Hoseini | Hoseini et al. 2025 | Brace | 17 | 5.39 | 1.53 | 43.58 | 6.14 | 5 (29%) | 12 (71%) | 5.2 | 10 | NO |
| Hoseini | Hoseini et al. 2025 | KT | 17 | 4.31 | 1.48 | 45 | 6.43 | 6 (35%) | 11 (65%) | 5.2 | 10 | NO |
| Hoseini | Hoseini et al. 2025 | CSI | 17 | 5.14 | 1.73 | 42 | 9.36 | 8 (47%) | 9 (53%) | 5.2 | 10 | NO |
| Akkurt | Akkurt et al. 2025 | KT | 21 | 6.0 (3–8) | 1.25 | 43 | 8.1 | 12 (57%) | 9 (43%) | 7 | 10 | NO |
| Akkurt | Akkurt et al. 2025 | Placebo | 21 | 5.0 (3.5-8) | 1.125 | 46.14 | 10 | 13 (62%) | 8 (38%) | 12 | 10 | NO |
| Kizilkurt | Kizilkurt et al. 2025 | CSI | 12 | 6.1 | 2.07 | 42.2 | 10.9 | 5 (42%） | 7 (58%） | 3 | 10 | NO |
| Kizilkurt | Kizilkurt et al. 2025 | PRP | 12 | 6.5 | 1.4 | 43.4 | 6.2 | 6 (50%) | 6 (50%) | 3 | 10 | NO |
| Kizilkurt | Kizilkurt et al. 2025 | Placebo | 12 | 7.2 | 1.3 | 40.7 | 9.4 | 5 (42%） | 7 (58%） | 3 | 10 | NO |

The table summarizes baseline characteristics of study arms included in the short-term network, including sample size, baseline pain severity, age, sex distribution, symptom duration, VAS scale, and combined-intervention status. These variables were examined as potential effect modifiers to assess the plausibility of the transitivity assumption across treatment comparisons. In the column “co”, YES indicates that the study arm involved a combined or multimodal intervention, whereas NO indicates a single intervention. NR indicates not reported. N, sample size; SD, standard deviation; VAS, visual analog scale; NR, not reported; Co, combined-intervention status.

Supplementary Table S2. Ranking probabilities and SUCRA values for short-term outcomes (1-4 weeks)

| Treatment | Rank 1 | Rank 2 | Rank 3 | Rank 4 | Rank 5 | Rank 6 | Rank 7 | Rank 8 | Rank 9 | Rank 10 | Rank 11 | Rank 12 | Rank 13 | Rank 14 | SUCRA |
| --- | --- | --- | --- | --- | --- | --- | --- | --- | --- | --- | --- | --- | --- | --- | --- |
| Brace | 0.01 | 0.04 | 0.07 | 0.11 | 0.14 | 0.14 | 0.12 | 0.11 | 0.09 | 0.07 | 0.05 | 0.02 | 0.01 | 0.00 | 57.13 |
| BT | 0.08 | 0.11 | 0.08 | 0.09 | 0.08 | 0.07 | 0.07 | 0.06 | 0.07 | 0.07 | 0.08 | 0.06 | 0.04 | 0.04 | 56.02 |
| CSI | 0.06 | 0.18 | 0.24 | 0.21 | 0.14 | 0.08 | 0.05 | 0.03 | 0.01 | 0.01 | 0.00 | 0.00 | 0.00 | 0.00 | 77.61 |
| DPT | 0.00 | 0.00 | 0.01 | 0.01 | 0.02 | 0.03 | 0.04 | 0.05 | 0.06 | 0.08 | 0.13 | 0.19 | 0.18 | 0.20 | 21.29 |
| ESWT | 0.00 | 0.00 | 0.01 | 0.04 | 0.09 | 0.13 | 0.16 | 0.17 | 0.16 | 0.12 | 0.07 | 0.03 | 0.01 | 0.00 | 46.97 |
| EX | 0.13 | 0.17 | 0.13 | 0.12 | 0.10 | 0.08 | 0.07 | 0.05 | 0.04 | 0.03 | 0.03 | 0.02 | 0.01 | 0.01 | 71.10 |
| GAGPS | 0.50 | 0.06 | 0.04 | 0.03 | 0.03 | 0.02 | 0.02 | 0.02 | 0.02 | 0.02 | 0.03 | 0.03 | 0.03 | 0.15 | 69.02 |
| KT | 0.18 | 0.33 | 0.25 | 0.13 | 0.06 | 0.03 | 0.01 | 0.01 | 0.00 | 0.00 | 0.00 | 0.00 | 0.00 | 0.00 | 86.39 |
| LA | 0.01 | 0.04 | 0.06 | 0.08 | 0.11 | 0.12 | 0.12 | 0.11 | 0.11 | 0.10 | 0.08 | 0.04 | 0.01 | 0.00 | 52.45 |
| Placebo | 0.00 | 0.00 | 0.00 | 0.00 | 0.00 | 0.00 | 0.00 | 0.00 | 0.01 | 0.03 | 0.08 | 0.24 | 0.39 | 0.24 | 10.01 |
| PRP | 0.00 | 0.01 | 0.01 | 0.01 | 0.02 | 0.03 | 0.03 | 0.04 | 0.06 | 0.08 | 0.12 | 0.17 | 0.18 | 0.24 | 20.43 |
| PT | 0.01 | 0.02 | 0.04 | 0.06 | 0.08 | 0.10 | 0.11 | 0.11 | 0.11 | 0.12 | 0.11 | 0.07 | 0.04 | 0.03 | 44.93 |
| Splint | 0.02 | 0.05 | 0.06 | 0.08 | 0.09 | 0.09 | 0.09 | 0.09 | 0.09 | 0.09 | 0.09 | 0.06 | 0.05 | 0.05 | 47.49 |
| US | 0.00 | 0.00 | 0.01 | 0.02 | 0.04 | 0.07 | 0.11 | 0.15 | 0.17 | 0.18 | 0.14 | 0.07 | 0.03 | 0.01 | 39.16 |

The table presents the ranking probabilities of each intervention across all possible ranks for short-term pain relief and the corresponding surface under the cumulative ranking curve (SUCRA) values. Higher SUCRA values indicate a greater probability that an intervention ranks among the more effective treatments; however, ranking results should be interpreted together with effect estimates, 95% credible intervals, and the amount of direct evidence.

Supplementary Table S3. Ranking probabilities and SUCRA values for intermediate-term outcomes (4-12 weeks)

| **Treatment** | **Rank 1** | **Rank 2** | **Rank 3** | **Rank 4** | **Rank 5** | **Rank 6** | **Rank 7** | **Rank 8** | **Rank 9** | **Rank 10** | **Rank 11** | **Rank 12** | **Rank 13** | **Rank 14** | **Rank 15** | **Rank 16** | **Rank 17** | **Rank 18** | **Rank 19** | **Rank 20** | **Rank 21** | **SUCRA** |
| --- | --- | --- | --- | --- | --- | --- | --- | --- | --- | --- | --- | --- | --- | --- | --- | --- | --- | --- | --- | --- | --- | --- |
| ASTM | 0.17 | 0.47 | 0.04 | 0.02 | 0.02 | 0.01 | 0.01 | 0.01 | 0.01 | 0.01 | 0.01 | 0.01 | 0.01 | 0.01 | 0.01 | 0.01 | 0.01 | 0.01 | 0.01 | 0.02 | 0.11 | 75.36 |
| Band | 0.00 | 0.03 | 0.07 | 0.06 | 0.06 | 0.05 | 0.05 | 0.04 | 0.04 | 0.04 | 0.04 | 0.04 | 0.04 | 0.05 | 0.05 | 0.05 | 0.05 | 0.05 | 0.05 | 0.06 | 0.06 | 46.65 |
| Brace | 0.00 | 0.02 | 0.05 | 0.07 | 0.07 | 0.08 | 0.08 | 0.07 | 0.07 | 0.07 | 0.06 | 0.06 | 0.06 | 0.06 | 0.05 | 0.04 | 0.04 | 0.03 | 0.02 | 0.01 | 0.00 | 55.71 |
| BT | 0.00 | 0.03 | 0.09 | 0.09 | 0.08 | 0.08 | 0.07 | 0.06 | 0.06 | 0.05 | 0.05 | 0.05 | 0.05 | 0.04 | 0.04 | 0.04 | 0.03 | 0.03 | 0.02 | 0.01 | 0.01 | 58.87 |
| CSI | 0.00 | 0.00 | 0.03 | 0.07 | 0.10 | 0.11 | 0.11 | 0.10 | 0.09 | 0.08 | 0.07 | 0.06 | 0.05 | 0.04 | 0.03 | 0.02 | 0.01 | 0.01 | 0.00 | 0.00 | 0.00 | 61.67 |
| CT | 0.00 | 0.04 | 0.08 | 0.06 | 0.05 | 0.04 | 0.04 | 0.03 | 0.03 | 0.03 | 0.03 | 0.03 | 0.03 | 0.04 | 0.04 | 0.04 | 0.05 | 0.05 | 0.06 | 0.08 | 0.13 | 42.41 |
| DPT | 0.00 | 0.00 | 0.00 | 0.01 | 0.01 | 0.02 | 0.02 | 0.03 | 0.04 | 0.05 | 0.06 | 0.07 | 0.08 | 0.09 | 0.10 | 0.11 | 0.11 | 0.10 | 0.07 | 0.04 | 0.02 | 33.81 |
| ESWT | 0.00 | 0.00 | 0.00 | 0.00 | 0.01 | 0.03 | 0.04 | 0.06 | 0.08 | 0.10 | 0.12 | 0.12 | 0.12 | 0.10 | 0.08 | 0.06 | 0.04 | 0.02 | 0.01 | 0.00 | 0.00 | 45.78 |
| EX | 0.00 | 0.02 | 0.06 | 0.08 | 0.07 | 0.07 | 0.06 | 0.06 | 0.06 | 0.05 | 0.05 | 0.05 | 0.05 | 0.05 | 0.05 | 0.05 | 0.05 | 0.04 | 0.04 | 0.03 | 0.01 | 52.75 |
| GAGPS | 0.80 | 0.15 | 0.01 | 0.01 | 0.00 | 0.00 | 0.00 | 0.00 | 0.00 | 0.00 | 0.00 | 0.00 | 0.00 | 0.00 | 0.00 | 0.00 | 0.00 | 0.00 | 0.00 | 0.00 | 0.00 | 97.24 |
| IONTO | 0.00 | 0.01 | 0.03 | 0.04 | 0.04 | 0.04 | 0.04 | 0.04 | 0.04 | 0.04 | 0.04 | 0.05 | 0.05 | 0.06 | 0.06 | 0.07 | 0.07 | 0.07 | 0.08 | 0.08 | 0.06 | 38.51 |
| KT | 0.01 | 0.17 | 0.36 | 0.19 | 0.11 | 0.06 | 0.04 | 0.02 | 0.02 | 0.01 | 0.01 | 0.00 | 0.00 | 0.00 | 0.00 | 0.00 | 0.00 | 0.00 | 0.00 | 0.00 | 0.00 | 84.83 |
| LA | 0.00 | 0.00 | 0.01 | 0.02 | 0.04 | 0.05 | 0.06 | 0.07 | 0.08 | 0.08 | 0.08 | 0.09 | 0.09 | 0.09 | 0.08 | 0.07 | 0.05 | 0.03 | 0.02 | 0.01 | 0.00 | 48.06 |
| MET | 0.00 | 0.01 | 0.02 | 0.02 | 0.02 | 0.02 | 0.02 | 0.02 | 0.02 | 0.03 | 0.03 | 0.03 | 0.03 | 0.04 | 0.04 | 0.05 | 0.06 | 0.07 | 0.09 | 0.13 | 0.25 | 25.07 |
| Placebo | 0.00 | 0.00 | 0.00 | 0.00 | 0.00 | 0.00 | 0.00 | 0.00 | 0.00 | 0.00 | 0.00 | 0.00 | 0.01 | 0.01 | 0.03 | 0.06 | 0.11 | 0.19 | 0.25 | 0.23 | 0.11 | 11.85 |
| PP | 0.00 | 0.01 | 0.02 | 0.03 | 0.03 | 0.03 | 0.03 | 0.03 | 0.03 | 0.04 | 0.04 | 0.04 | 0.05 | 0.05 | 0.06 | 0.07 | 0.08 | 0.08 | 0.09 | 0.11 | 0.10 | 32.59 |
| PRP | 0.00 | 0.00 | 0.00 | 0.00 | 0.01 | 0.01 | 0.01 | 0.02 | 0.02 | 0.02 | 0.03 | 0.04 | 0.04 | 0.05 | 0.07 | 0.08 | 0.10 | 0.12 | 0.13 | 0.13 | 0.11 | 22.63 |
| PT | 0.00 | 0.01 | 0.03 | 0.06 | 0.08 | 0.08 | 0.09 | 0.09 | 0.08 | 0.08 | 0.07 | 0.07 | 0.06 | 0.06 | 0.05 | 0.04 | 0.03 | 0.02 | 0.01 | 0.01 | 0.00 | 55.93 |
| PU | 0.00 | 0.03 | 0.08 | 0.10 | 0.09 | 0.09 | 0.08 | 0.07 | 0.07 | 0.06 | 0.06 | 0.05 | 0.05 | 0.04 | 0.04 | 0.03 | 0.03 | 0.02 | 0.01 | 0.01 | 0.00 | 60.71 |
| Splint | 0.00 | 0.00 | 0.02 | 0.04 | 0.04 | 0.05 | 0.05 | 0.05 | 0.05 | 0.06 | 0.06 | 0.06 | 0.06 | 0.07 | 0.07 | 0.07 | 0.07 | 0.06 | 0.05 | 0.04 | 0.02 | 43.16 |
| US | 0.00 | 0.00 | 0.01 | 0.04 | 0.07 | 0.09 | 0.10 | 0.10 | 0.10 | 0.09 | 0.08 | 0.08 | 0.07 | 0.05 | 0.04 | 0.03 | 0.02 | 0.01 | 0.00 | 0.00 | 0.00 | 56.42 |

The table presents the ranking probabilities of each intervention across all possible ranks for intermediate-term pain relief and the corresponding surface under the cumulative ranking curve（SUCRA）values. Higher SUCRA values indicate a greater probability that an intervention ranks among the more effective treatments; however, ranking results should be interpreted together with effect estimates, 95% credible intervals, and the amount of direct evidence.

Supplementary Table S4. Ranking probabilities and SUCRA values for long-term outcomes (＞12 weeks)

| Treatment | Rank 1 | Rank 2 | Rank 3 | Rank 4 | Rank 5 | Rank 6 | Rank 7 | Rank 8 | Rank 9 | SUCRA |
| --- | --- | --- | --- | --- | --- | --- | --- | --- | --- | --- |
| CSI | 0.00 | 0.00 | 0.00 | 0.00 | 0.01 | 0.05 | 0.12 | 0.26 | 0.55 | 9.01 |
| ESWT | 0.00 | 0.01 | 0.04 | 0.09 | 0.15 | 0.22 | 0.24 | 0.18 | 0.07 | 33.56 |
| GAGPS | 0.62 | 0.11 | 0.03 | 0.02 | 0.01 | 0.01 | 0.01 | 0.02 | 0.18 | 75.93 |
| HVLAT | 0.06 | 0.30 | 0.43 | 0.12 | 0.04 | 0.02 | 0.01 | 0.01 | 0.01 | 75.56 |
| MET | 0.01 | 0.09 | 0.25 | 0.31 | 0.13 | 0.10 | 0.06 | 0.03 | 0.01 | 60.24 |
| Placebo | 0.00 | 0.00 | 0.01 | 0.04 | 0.12 | 0.22 | 0.23 | 0.32 | 0.05 | 27.67 |
| PRP | 0.30 | 0.45 | 0.07 | 0.04 | 0.03 | 0.02 | 0.02 | 0.03 | 0.05 | 79.14 |
| PT | 0.00 | 0.01 | 0.08 | 0.24 | 0.34 | 0.17 | 0.13 | 0.02 | 0.00 | 49.46 |
| Splint | 0.01 | 0.03 | 0.08 | 0.14 | 0.17 | 0.19 | 0.17 | 0.14 | 0.08 | 39.42 |

The table presents the ranking probabilities of each intervention across all possible ranks for long-term pain relief and the corresponding surface under the cumulative ranking curve (SUCRA) values. Higher SUCRA values indicate a greater probability that an intervention ranks among the more effective treatments; however, ranking results should be interpreted together with effect estimates, 95% credible intervals, and the amount of direct evidence.
