# Supplementary material for: Time-dependent comparative efficacy of non-surgical treatments for pain relief in lateral epicondylitis: a systematic review and network meta-analysis
Source: Front Physiol. 2026 Mar 27;17:1782562. doi: 10.3389/fphys.2026.1782562 (PMC13066320; doi:10.3389/fphys.2026.1782562)
Supplement: Supplementary file 2 [file Table1.docx]

**Supplementary Table S5. Detailed search strategies**

**Time-Dependent Comparative Efficacy of Non-Surgical Treatments for Pain Relief in Lateral Epicondylitis: A Systematic Review and Network Meta-Analysis**

Note. The table presents the detailed search strategies used for PubMed, Web of Science, and Cochrane Library, including the search concepts, exact search strings, and the numbers of records retrieved in the initial and updated searches. Search terms were grouped into population, intervention, and study design domains. "Laser therapy" is used as the corrected wording for the original entry "lazer therapy".

**Search concepts**

| **Concept** | **Terms** |
| --- | --- |
| Population | Tennis Elbow; Lateral Epicondylitis; Lateral Epicondylalgia |
| Intervention | Conservative Treatments; Electrophysiotherapy; Physiotherapy; Physical Therapy; Extracorporeal Shock Wave Therapy; Injections; Platelet Rich Plasma (PRP) Injections; Rehabilitation; Acupuncture; Kinesitherapy; Rest; Cryotherapy; Ultrasound; Laser Therapy; Manual Therapy; Exercises Strengthening; Splint |
| Study design | RCT; Clinical Trials |

**PubMed**

Initial search: 863 records; updated search: 23 records.

**#1 Population search:** ((Tennis Elbow[Title/Abstract]) OR (Lateral Epicondylitis[Title/Abstract])) OR (Lateral Epicondylalgia[Title/Abstract])

**Alternative display:** "tennis elbow"[Title/Abstract] OR "lateral epicondylitis"[Title/Abstract] OR "lateral epicondylalgia"[Title/Abstract]

**#2 Intervention search:** ((((((((((((((((((Conservative Treatments[Title/Abstract]) OR (Electrophysiotherapy[Title/Abstract])) OR (Physiotherapy[Title/Abstract])) OR (Physical Therapy[Title/Abstract])) OR (Extracorporeal Shock Wave Therapy[Title/Abstract])) OR (Injections[Title/Abstract])) OR (Platelet Rich Plasma (PRP) Injections[Title/Abstract])) OR (Rehabilitation[Title/Abstract])) OR (Acupuncture[Title/Abstract])) OR (Kinesitherapy[Title/Abstract])) OR (Rest[Title/Abstract])) OR (Cryotherapy[Title/Abstract])) OR (Ultrasound[Title/Abstract])) OR (Laser Therapy[Title/Abstract])) OR (Manual therapy[Title/Abstract])) OR (Exercises Strengthening[Title/Abstract])) OR (Splint[Title/Abstract])

**Alternative display:** "conservative treatments"[Title/Abstract] OR "Electrophysiotherapy"[Title/Abstract] OR "Physiotherapy"[Title/Abstract] OR "physical therapy"[Title/Abstract] OR "extracorporeal shock wave therapy"[Title/Abstract] OR "Injections"[Title/Abstract] OR ((("platelet rich plasma"[MeSH Terms] OR ("platelet rich"[All Fields] AND "plasma"[All Fields]) OR "platelet rich plasma"[All Fields] OR ("platelet"[All Fields] AND "rich"[All Fields] AND "plasma"[All Fields]) OR "platelet rich plasma"[All Fields]) AND ("pharmacol res perspect"[Journal] OR "prp"[All Fields])) AND "Injections"[Title/Abstract]) OR "Rehabilitation"[Title/Abstract] OR "Acupuncture"[Title/Abstract] OR "Kinesitherapy"[Title/Abstract] OR "Rest"[Title/Abstract] OR "Cryotherapy"[Title/Abstract] OR "Ultrasound"[Title/Abstract] OR ("laser"[All Fields] AND "Therapy"[Title/Abstract]) OR "manual therapy"[Title/Abstract] OR "exercises strengthening"[Title/Abstract] OR "Splint"[Title/Abstract]

**Translations:** Platelet Rich Plasma: "platelet-rich plasma"[MeSH Terms] OR ("platelet-rich"[All Fields] AND "plasma"[All Fields]) OR "platelet-rich plasma"[All Fields] OR ("platelet"[All Fields] AND "rich"[All Fields] AND "plasma"[All Fields]) OR "platelet rich plasma"[All Fields]
PRP: "Pharmacol Res Perspect"[Journal:__jid101626369] OR "prp"[All Fields]

**#3 Final combination:** (((Tennis Elbow[Title/Abstract]) OR (Lateral Epicondylitis[Title/Abstract])) OR (Lateral Epicondylalgia[Title/Abstract])) AND (((((((((((((((((Conservative Treatments[Title/Abstract]) OR (Electrophysiotherapy[Title/Abstract])) OR (Physiotherapy[Title/Abstract])) OR (Physical Therapy[Title/Abstract])) OR (Extracorporeal Shock Wave Therapy[Title/Abstract])) OR (Injections[Title/Abstract])) OR (Platelet Rich Plasma (PRP) Injections[Title/Abstract])) OR (Rehabilitation[Title/Abstract])) OR (Acupuncture[Title/Abstract])) OR (Kinesitherapy[Title/Abstract])) OR (Rest[Title/Abstract])) OR (Cryotherapy[Title/Abstract])) OR (Ultrasound[Title/Abstract])) OR (Laser Therapy[Title/Abstract])) OR (Manual therapy[Title/Abstract])) OR (Exercises Strengthening[Title/Abstract])) OR (Splint[Title/Abstract]))

**Alternative display:** ("tennis elbow"[Title/Abstract] OR "lateral epicondylitis"[Title/Abstract] OR "lateral epicondylalgia"[Title/Abstract]) AND ("conservative treatments"[Title/Abstract] OR "Electrophysiotherapy"[Title/Abstract] OR "Physiotherapy"[Title/Abstract] OR "physical therapy"[Title/Abstract] OR "extracorporeal shock wave therapy"[Title/Abstract] OR "Injections"[Title/Abstract] OR ((("platelet rich plasma"[MeSH Terms] OR ("platelet rich"[All Fields] AND "plasma"[All Fields]) OR "platelet rich plasma"[All Fields] OR ("platelet"[All Fields] AND "rich"[All Fields] AND "plasma"[All Fields]) OR "platelet rich plasma"[All Fields]) AND ("pharmacol res perspect"[Journal] OR "prp"[All Fields])) AND "Injections"[Title/Abstract]) OR "Rehabilitation"[Title/Abstract] OR "Acupuncture"[Title/Abstract] OR "Kinesitherapy"[Title/Abstract] OR "Rest"[Title/Abstract] OR "Cryotherapy"[Title/Abstract] OR "Ultrasound"[Title/Abstract] OR ("laser"[All Fields] AND "Therapy"[Title/Abstract]) OR "manual therapy"[Title/Abstract] OR "exercises strengthening"[Title/Abstract] OR "Splint"[Title/Abstract])

**Web of Science**

Initial search: 853 records; updated search: 99 records.

**#1 Population search:** ((AB=(Tennis Elbow)) OR AB=(Lateral Epicondylitis)) OR AB=(Lateral Epicondylalgia)

**#2 Intervention search:** ((((((((((((((((((AB=(Conservative Treatments)) OR AB=(Electrophysiotherapy)) OR AB=(Physiotherapy)) OR AB=(Physical Therapy)) OR AB=(Extracorporeal Shock Wave Therapy)) OR AB=(Injections)) OR AB=(Platelet Rich Plasma (PRP) Injections)) OR AB=(Rehabilitation)) OR AB=(Acupuncture)) OR AB=(Kinesitherapy)) OR AB=(Rest)) OR AB=(Cryotherapy)) OR AB=(Ultrasound)) OR AB=(Laser Therapy)) OR AB=(Manual therapy)) OR AB=(Exercises Strengthening)) OR AB=(Splint)

**#3 Final combination:** #1 AND #2

**Cochrane Library**

Initial search: 1042 records; updated search: 220 records.

**#1 Population search:** [Title Abstract Keyword] Tennis Elbow OR Lateral Epicondylitis OR Lateral Epicondylalgia

**#2 Intervention search:** [Title Abstract Keyword] Conservative Treatments OR Electrophysiotherapy OR Physiotherapy OR Physical Therapy OR Extracorporeal Shock Wave Therapy OR Injections OR Platelet Rich Plasma (PRP) Injections OR Rehabilitation OR Acupuncture OR Kinesitherapy OR Rest OR Cryotherapy OR Ultrasound OR Laser Therapy OR Manual therapy OR Exercises Strengthening OR Splint

**#3 Final combination:** #1 AND #2
